# Advancing Brain Tumor Diagnosis Using Deep Learning: A Systematic and Critical Review on Methodological Approaches to Glioma Segmentation and Classification Through Multiparametric MRI

**DOI:** 10.3390/brainsci16050468

**Published:** 2026-04-27

**Authors:** Simona Aresta, Cinzia Palmirotta, Muhammad Asim, Petronilla Battista, Gaia C. Santi, Gianvito Lagravinese, Claudia Cava, Pietro Fiore, Andrea Santamato, Paolo Vitali, Isabella Castiglioni, Gennaro D’Anna, Leonardo Rundo, Christian Salvatore

**Affiliations:** 1 Ailice Labs, Department of Science, Technology and Society, University School for Advanced Studies IUSS Pavia, 27100 Pavia, Italy; muhammad.asim@iusspavia.it (M.A.); claudia.cava@iusspavia.it (C.C.); christian.salvatore@iusspavia.it (C.S.); 2 Istituti Clinici Scientifici Maugeri IRCCS, Laboratory of Neuropsychology, Institute of Bari, 70124 Bari, Italygaia.santi@icsmaugeri.it (G.C.S.); gianvito.lagravinese@icsmaugeri.it (G.L.); 3 Istituti Clinici Scientifici Maugeri IRCCS, Neurorehabilitation Unit of Bari Institute, 70124 Bari, Italy; pietro.fiore@icsmaugeri.it; 4 Department of Physical and Rehabilitation Medicine, University of Foggia, 71122 Foggia, Italy; 5 Spasticity and Movement Disorders “ReSTaRt”, Physical Medicine and Rehabilitation Section, Department of Medical and Surgical Sciences, University of Foggia, 71122 Foggia, Italy; andrea.santamato@unifg.it; 6 Department of Biomedical Sciences for Health, Università degli Studi di Milano, 20122 Milan, Italy; paolo.vitali@unimi.it; 7 Unit of Radiology, IRCCS Galeazzi-Sant’Ambrogio Hospital, 20157 Milan, Italy; 8 Department of Physics ‘‘Giuseppe Occhialini”, University of Milan-Bicocca, 20126 Milan, Italy; isabella.castiglioni@unimib.it; 9 CDI Centro Diagnostico Italiano, 20147 Milan, Italy; 10 Department of Diagnostic Imaging and Stereotactic Radiosurgery, Centro Diagnostico Italiano S.p.A., 20147 Milan, Italy; gennaro.danna@gmail.com; 11 Department of Information Engineering, Electrical Engineering and Applied Mathematics (DIEM), University of Salerno, 84084 Fisciano, Italy; lrundo@unisa.it; 12 DeepTrace Technologies S.R.L., 20122 Milan, Italy

**Keywords:** brain tumors, glioma, segmentation, classification, deep learning, medical imaging

## Abstract

**Highlights:**

**What are the main findings?**

- In total, 31 of 310 studies (2022–2025) met the inclusion criteria; eight did both segmentation and classification.
- CNN-based (especially U-Net variants) and hybrid models dominate glioma segmentation and classification, but clinical translation remains limited due to a lack of external validation, dataset bias, and heterogeneous methodologies.

**What are the implications of the main findings?**

- Future research should prioritize cross-institutional validation on independent clinical cohorts and the adoption of standardized evaluation and reporting protocols to improve reliability and comparability.
- Integrating imaging with clinical and molecular data, together with the systematic use of explainability methods, will be essential to develop robust and clinically deployable AI models.

**Abstract:**

**Background/Objectives:** Brain tumors are highly lethal cancers, with gliomas representing the most complex subtype. Magnetic resonance imaging (MRI) is the main non-invasive imaging modality. This review evaluates deep learning (DL) and artificial intelligence methods for brain tumor segmentation and classification. **Methods:** In this systematic review, PubMed and Scopus were searched for articles published from 2022 to March 2025. Authors independently identified eligible studies based on predefined inclusion criteria and extracted data. The study quality and risk of bias were assessed using the Quality Assessment of Diagnostic Accuracy Studies (QUADAS) checklist. **Results:** Thirty-one studies met the inclusion criteria from 310 records, with eight addressing both segmentation and classification. Most segmentation studies used publicly available multiparametric MRI datasets. Performance varied by architecture and tumor region, with whole-tumor segmentation achieving the highest Dice Similarity Coefficient (DSC). Classical U-Nets reported DSC values ranging 80–87%, while models with residual or attention mechanisms exceeded 90%. Classification focused on tumor type and glioma grading, using features learned from multiparametric MRI. Reported accuracy ranged from 91.3% to 99.4%, with sensitivity and specificity often above 95%. However, variability across tumor subregions, limited external validation, reliance on public datasets, and heterogeneous preprocessing raise concerns about robustness and real-world generalizability. Evidence on the use of explainability methods for both tasks remains limited. **Conclusions:** DL models for glioma segmentation and classification demonstrate promising performance. However, standardized validation protocols, multi-center datasets, and the integration of explainable artificial intelligence techniques are needed to improve transparency, robustness, and clinical applicability.

## 1. Introduction

Malignant brain tumors, although relatively rare, are one of the most lethal forms of cancer [1]. In 2022, Bray and colleagues [2] identified brain tumors as the 19th in the incidence rank, with 321,476 cases, and the 12th in the mortality rank, with 248,305 deaths. Brain tumors can be broadly categorized as primary or secondary. The three most common types of primary brain tumors are meningioma, glioma, and pituitary tumors [3]. Among these, gliomas are the most common malignant ones, with glioblastoma being the most prevalent and aggressive subtype.

Gliomas originate from glial cells and represent a heterogeneous group of tumors, ranging from low-grade gliomas (LGG) to very aggressive forms, classified as high-grade gliomas (HGG; i.e., glioblastoma) [4,5]. LGGs grow slowly, whereas HGGs grow rapidly and are sometimes untreatable, even with advanced techniques. Gliomas may exhibit either well-circumscribed boundaries or diffusely infiltrative growth patterns, making segmentation and classification particularly challenging [6]. After tumor detection, an accurate diagnostic pipeline requires both tumor segmentation and classification before any therapeutic intervention.

To this end, among neuroimaging techniques, positron emission tomography (PET) measures the metabolic activity of brain tumors, and magnetic resonance imaging (MRI) provides structural information [7]. MRI is considered the gold standard due to its non-invasive nature and ability to provide high soft-tissue contrast and spatial resolution [8,9]. Four standard MRI sequences are used for glioma diagnosis: T1-weighted MRI (T1), for discriminating healthy tissues and detect intralesional hemorrhages; T2-weighted MRI (T2), to delineate the edema region; T1-weighted MRI with gadolinium contrast enhancement (T1ce), to outline the more malignant part of the tumor; and Fluid-Attenuated Inversion Recovery (FLAIR), which suppresses cerebrospinal fluid signal, is particularly useful in glioma imaging, especially in glioblastoma, for highlighting abnormalities and assisting in the differentiation between vasogenic edema and infiltrative tumor, although this distinction remains challenging [10]. Therefore, a Multiparametric MRI (mpMRI) scan is the most suitable approach for segmenting gliomas.

Glioma segmentation is essential for removing confounding structures and supporting accurate diagnosis. It delineates brain tumors anatomically, distinguishing neoplastic regions from healthy tissue and thereby requiring different treatment approaches. It also enables longitudinal MRI monitoring to track tumor recurrence, growth, or shrinkage [11,12]. Glioma segmentation can be particularly challenging due to several factors, e.g., intensity and poorly defined boundaries on MRI [13,14], interpatient variability in size, spatial extent, and anatomical location [15]. Moreover, the presence of imaging artifacts and noise in MRI also increases the difficulty in segmentation [16].

Several glioma subregions are clinically relevant and must be segmented: (i) core tumor (CT), defined as the combination of the non-enhancing tumor (NET), edema (ED), and necrotic regions (NCR) [17,18], represents the central area containing the most aggressive cells, and is crucial for assessing tumor severity and guiding treatment and (ii) the enhancing tumor (ET), marked by active growth and increased vascularity, essential for understanding progression, planning surgery or radiotherapy, and estimating survival. Accurate segmentation of these regions is vital [7]. Segmentation techniques can be classified, according to the level of human interaction required, into manual, semi-automatic, and fully automatic methods [19,20,21]. Manual segmentation is time-consuming, requires expert knowledge, and is prone to operator variability [22]. Semi-automatic approaches require user input for initialization or correction [22], whereas fully automatic methods operate without human intervention, typically relying on model-based techniques that incorporate prior knowledge of tumor characteristics such as shape, size, appearance, and location [22].

Glioma classification is essential to ensure an accurate and reproducible identification of glioma subtypes [5]. Histopathological classification is labor-intensive, requiring manual examination of both coarse and fine microscope image resolutions across large tissue volumes. When multiple pathologists classify the same sample, inconsistencies can arise due to subjective perception. Interobserver variability is a significant challenge, as prognostic outcomes can differ even within a single glioma subtype [23].

Artificial Intelligence (AI) and radiomics have emerged as complementary tools for glioma characterization, with increasing emphasis on developing explainable pipelines to facilitate clinical translation [24,25]. AI supports tumor segmentation and classification through machine learning (ML) algorithms that analyze radiomic features and deep learning (DL) models that automatically segment tumors and extract complex imaging patterns [26,27]. Radiomics enables non-invasive analysis by extracting quantitative features from medical images, i.e., shape, texture, intensity, and heterogeneity, thereby supporting glioma classification alongside histopathology [28,29], as well as prognosis and informing treatment planning [30,31]. In particular, radiomics can help determine the risk of disease progression after treatment [30] and select the best treatment options by predicting important molecular markers, such as O^6^-methylguanine-DNA methyltransferase (MGMT) promoter methylation status [31], from related imaging patterns without requiring invasive procedures. DL, a subfield of ML, uses large neural networks to learn hierarchical representations directly from data, often achieving performance comparable to or exceeding traditional ML methods in medical image analysis [27,32]. Most state-of-the-art approaches rely on convolutional neural networks (CNNs), which automatically learn relevant features from imaging data for tasks such as tumor segmentation and classification [32,33]. Among these, U-Net is the most widely used encoder–decoder CNN architecture for medical image segmentation, and its latent features can also support classification tasks [34].

This systematic and critical review aims to critically evaluate and summarize the current state-of-the-art of DL-based approaches for glioma segmentation as the primary objective, and, where applicable, to further analyze studies that also address glioma classification as a subsequent step in the diagnostic pipeline. To this end, we established specific inclusion criteria to select relevant studies primarily focused on segmentation, and, among these, we identified and examined those that integrated a classification task. We then highlighted and compared their main characteristics, including the datasets used, imaging modalities, DL architecture, and segmentation and classification performance. Finally, we discuss the significance of achieving precise and accurate tumor segmentation as a prerequisite for reliable classification.

## 2. Materials and Methods

### 2.1. Search and Selection Criteria

This review was conducted on published papers that apply DL algorithms to the automatic segmentation of glioma in medical imaging. A study protocol outlining the search strategy and review methodology was preregistered in the Open Science Framework (OSF) database on 2 December 2025 (Aresta S, Palmirotta C, Salvatore C, Asim M. Advancing Brain Tumor Diagnosis Using Deep Learning: A Systematic and Critical Review on Methodological Approaches to Glioma Segmentation and Classification through Multiparametric MRI. OSF Registration. Available at: https://osf.io/v54hs, accessed on 10 March 2026). This work was performed and reported following Preferred Reporting Items for Systematic Reviews and Meta-Analyses (PRISMA) guidelines [35] (see the PRISMA checklist in the Supplementary Materials). To identify studies for inclusion in the review, the PICOS approach was used. Criteria for study inclusion and exclusion were established prior to the review. Papers were considered for inclusion only if (i) they were written in full-text English language in a peer-reviewed journal; (ii) they were published prior to our search on 14 March 2025; (iii) they were published from 1 January 2022 to 14 March 2025; (iv) they employed a DL algorithm for the segmentation task; (v) they specifically targeted the segmentation of glioma brain tumors; and (vi) they were fully accessible in full-text format. Studies were excluded if they met any of the following criteria: (a) they did not report segmentation performance metrics such as Dice Similarity Coefficient (DSC) or Intersection over Union (IoU); (b) they addressed only tumor detection or classification without segmentation; (c) they used AI-generated tumor masks as ground truth for training; (d) they were book chapters, review articles, surveys, or conference proceedings or (e) they were published before 1 January 2022. First, two authors independently screened the publications for the review (SA, CS). Papers that satisfied all predefined criteria were ultimately included in the review. After identifying the included papers, two authors (MA and SA) independently reviewed them to determine whether they performed a classification task. Studies were considered eligible for classification analysis only if they (i) used an AI algorithm to perform the classification task and (ii) reported classification performance metrics, such as accuracy. Any discrepancies between the reviewers were resolved through discussion and consensus.

### 2.2. Information Source and Search

A comprehensive search strategy was developed in collaboration with a librarian, incorporating both keywords and Medical Subject Headings (MeSH). One author (SA) conducted a literature search in PubMed and Scopus. The search was conducted on 14 March 2025. The search strategy based on the PICOS method was applied following five concepts: (1) Population, defined as patients with glioma; (2) Intervention, defined as DL algorithms for glioma segmentation; (3) Comparison, defined as the manual segmentation of glioma; (4) Outcome, defined as the segmentation performance; (5) Study design, which should be “cross-sectional” or “longitudinal studies”. The search strategy was formed around four concepts: “brain tumor,” “glioma,” “segmentation,” and “deep learning”. Synonyms within each concept were combined with the OR Boolean operator, and terms between concepts were combined with the AND Boolean operator. The following keywords (with both extended names and abbreviations) were used for the literature search: ((“automatic segmentation” OR “segmentation”) AND (“deep learning” OR “DL”) AND (“brain” AND (“tumor” OR “tumor”)) AND (“detection”) AND (“glioma”)). The authors did not manually search reference lists from previously retrieved articles.

### 2.3. Study Selection

Study selection was performed independently by two reviewers (SA, CP). After the deduplication procedure, the titles of the retrieved studies were initially screened, followed by abstract screening conducted by one reviewer (SA), who assessed abstracts for eligibility. The second reviewer (CP) independently revised the list of potential articles based on abstracts. Articles deemed potentially eligible were subsequently reviewed in detail by the same reviewers for quality assessment. Full-text evaluations were conducted to exclude studies that did not meet the inclusion criteria when eligibility could not be determined from the title and abstract alone. All studies reporting data suitable for pooling were included in the quantitative analysis. Specifically, we limited the analysis to studies that reported at least one performance metric of the automatic segmentation, including DSC or IoU.

From this pool of eligible studies, two reviewers (MA, SA) independently assessed, in full-text evaluations, whether the studies performed a classification task in addition to segmentation. Specifically, we included only studies in the classification analysis that reported at least one performance metric focused on tumor classification.

### 2.4. Data Extraction Strategy

The data extracted from each study were organized into the following categories: study population, sample size and dataset used, imaging modality, image preprocessing techniques, postprocessing techniques for segmentation masks, validation strategy, use of external validation datasets, DL model employed for segmentation, model hyperparameters, segmented tumor regions, performance metrics used for model evaluation, use of explainable AI (XAI) methods, and reported performance results, i.e., study-specific DSC and study-specific IoU.

For studies that also performed the classification task, we additionally extracted: feature extraction method, feature used, feature selection, validation strategy, external validation, AI model for classification, classification hyperparameters, classes considered, performance metrics used for model evaluation, any use of an XAI method, and reported performance results (i.e., study-specific accuracy).

### 2.5. Risk of Bias in Individual Studies

In accordance with Cochrane guidelines, the Quality Assessment of Diagnostic Accuracy Studies (QUADAS) tool [36] was employed to evaluate the methodological quality and risk of bias in each included study. Furthermore, the same tool was used for each paper included in the classification pool.

Although QUADAS-2 was originally developed for diagnostic accuracy studies, it was adapted in this review to account for AI-based methodologies. In particular, patient selection was interpreted in terms of dataset provenance and sampling strategy; the index test domain was mapped to model development and validation procedures (including train–test separation and cross-validation); and the reference standard domain was evaluated based on annotation procedures and expert involvement. Flow and timing were assessed with respect to data splits and inclusion of all available samples in the final analysis.

Based on this assessment, studies were categorized as having low, high, or unclear risk of bias. Only studies classified as high quality were included in the subgroup analyses for the meta-analysis.

## 3. Results

### 3.1. Study Selection

**Figure 1:**
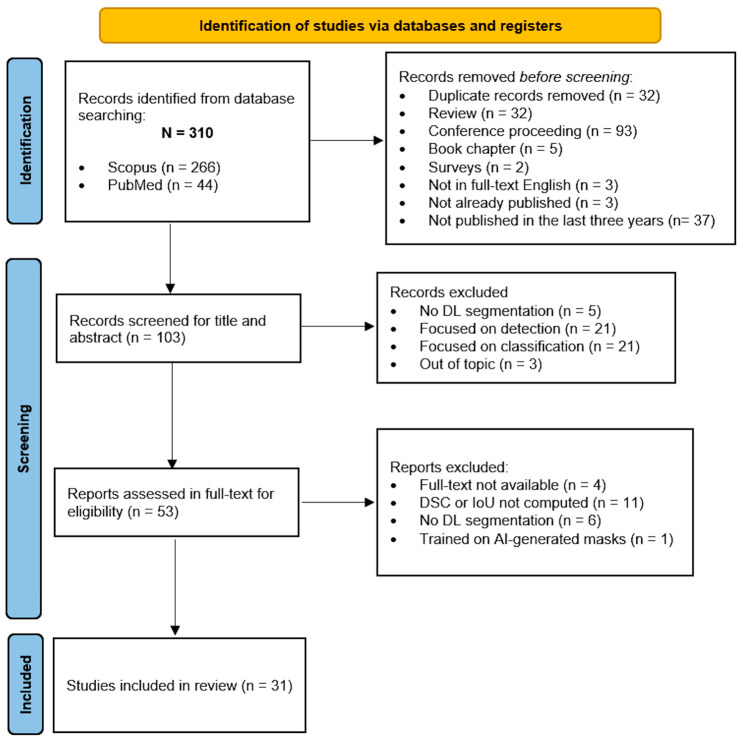
PRISMA flow chart outlining the sequential stages of the review selection process.

All the stages of the selection process are shown in the PRISMA flow diagram in Figure 1. The literature search yielded 310 papers from two electronic databases; no additional papers were included in the list of previously retrieved papers. Before screening, 207 papers were excluded: 32 duplicate records; 32 reviews; 93 conference proceedings (i.e., conference papers and reviews); five book chapters, two surveys; three papers not written in full-text English (i.e., one written in Persian and two written in Chinese), two papers not already published in peer-reviewed journals; and 37 papers published before 2022. A total of 104 papers were screened based on titles and abstracts. 50 papers were excluded after the abstract and title screening. In particular, (i) five studies were excluded because they performed segmentation not using DL algorithms; (ii) 21 studies focused on detection of brain-tumor mass; (iii) 21 studies focused only on classification of brain tumors phenotypes; and (iv) three papers were out this review topic, one describing a quantitative system designed to standardize glioma imaging descriptions, another one mainly explaining theoretically EXplainable AI (XAI), and the latter focusing on the impact of two major programming environments on the accuracy of DL models for glioma detection. Therefore, at this screening phase, 53 papers were assessed in full-text for eligibility. At this step, 22 papers were excluded: (a) four papers were not available in full text, even after the authors’ request; (b) 11 papers did not provide DSC or IoU as segmentation performance metrics, but in the majority of the cases they computed only accuracy; (c) six papers performed segmentation not using DL methods, favoring the implementation of unsupervised clustering techniques, Wavelet filters, or region-growing algorithms; and (d) one paper was aimed at using annotation by clinicians and a Transformer-based model to generate the brain tumor segmentation masks to further train a Generative Adversarial Network (GAN) through a self-supervised approach. In the final stage, 31 studies met the eligibility criteria and were included in the review.

These papers were further screened to analyze how many not only performed segmentation using DL algorithms but also performed a classification task using AI and reported performance metrics. Only eight studies met this second eligibility criterion of classification and were further analyzed.

### 3.2. Results of the Systematic and Critical Review

#### 3.2.1. Brain Tumor Segmentation

Table 1 summarizes the studies included in the review and provides detailed information on the study population, tumor types under examination, dataset used, sample size, imaging modality for the segmentation task, image preprocessing techniques, segmentation mask postprocessing, external validation or fine-tuning datasets, validation strategies for model training and testing, the DL model architecture, its hyperparameters, performance metrics used for evaluation (e.g., DSC, IoU, accuracy), and the application of any XAI methods. The major findings regarding model performance are presented in Table 2. Performance metrics are better explained in the Supplementary Materials (see Table S1).

The final search yielded 31 studies. While most of these studies focused only on patients, three also included healthy controls (HC) [37,38,39]. One study [37] focused specifically on the segmentation of pediatric brain tumors using DL algorithms. Three studies investigated segmentation for treatment-response assessment: two explored surgical treatment, and one oral chemotherapy. Specifically, Bouget et al. (2022) [40] trained a DL model using pre-operative MRI to segment brain tumors, aiming to improve the assessment of tumor size, location, and involvement of surrounding brain structures to optimize surgical planning. Zanier et al. (2023) [41] developed a DL-based segmentation model trained on pre-operative MRI and fine-tuned on post-operative MRI to automatically compute tumor volume and estimate the extent of resection. In addition, Gutsche et al. (2023) [42] trained a DL algorithm on 18F-fluoroethyl-L-tyrosine PET (18F-FET PET) images from patients treated with adjuvant temozolomide following surgery to segment the Metabolic Tumor Volume (MTV) and predict disease prognosis and overall survival.

Among the selected studies, 21 focused exclusively on gliomas [17,18,40,41,43,44,45,46,47,48,49,50,51,52,53,54,55,56,57,58,59]. Of these, four specifically addressed the segmentation of LGG only [48,56,58,59], while the remaining studies included both LGG and HGG. A smaller subset of studies (eight out of 31) also included other brain tumor types, such as meningiomas and pituitary tumors [38,39,60,61,62,63,64,65]. Furthermore, two studies [37,42] focused on a large variety of brain tumors and metastasis.

The majority of the papers used publicly available datasets, in particular, Brain Tumor Segmentation (BraTS) [15,66,67], Brain Tumor Figshare (BTF) [68,69], and The Cancer Genome Atlas Low Grade Glioma (TCGA-LGG) [70]. The BraTS dataset is a widely used, curated collection of multiparametric 3D brain MRIs from patients with both LGG and HGG. Each scan includes expert-approved manual segmentations of tumor subregions (whole tumor (WT), CT, ET), annotated by up to four raters following a standardized protocol and checked by experienced neuroradiologists. All images are preprocessed, i.e., co-registered, skull-stripped, resampled to 1 mm^3^ resolution, and intensity-normalized, for consistency and ease of use. A new BraTS dataset has been released every year from 2012 to the present, continuously expanding the number of subjects, improving data quality, and adding clinical information to support research on brain tumors. The BTF dataset includes T1ce-weighted MRIs of glioma, meningioma, and pituitary tumors, coupled with a binary mask indicating the WT, generated as the average segmentation from three experienced raters. The TCGA-LGG dataset contains mpMRI of LGG, coupled with WT segmentation generated using a semi-automated method, visually checked and modified as necessary by an experienced neuroradiologist. Delving into the included studies, 13 used only the BraTS datasets from 2017 to 2021 [17,18,43,46,47,49,50,51,52,53,54,55,56,57], four used only the BTF dataset [60,61,62,63], and three used only the TCGA-LGG [48,58,59]. Furthermore, three papers used a combination of these datasets [44,64,65]. Other less prominent datasets from Kaggle were also used in a small subset, such as the Kaggle Brain Tumor MRI dataset [71] and the Kaggle Brain Classification dataset [72], either independently [38] or in combination with BraTS datasets [39]. In particular, one study [52] used the BraTS datasets in combination with the UPENN-GBM dataset [73]. Additionally, five studies used custom-made datasets independently [37,42] or in combination with BraTS datasets [40,41,45].

With regard to population size, studies relied either on a single dataset or on multiple datasets. In the latter case, the total number of patients was computed as the sum of the patient counts across the datasets. Based on this approach, the median number of patients was 369, with a range of 65 to 3264.

Looking at the image modalities, only one paper used 18F-FET PET images [27]; the other 30 papers used MRI. Specifically, seven studies used T1ce-weighted MRI [38,60,61,62,63,64,65], three studies used FLAIR MRI images [48,58,59], 13 studies used FLAIR, T1-weighted, T1ce-weighted and T2-weighted MRI [17,18,39,43,44,47,50,51,52,53,54], one used FLAIR, T1-weighted, T1ce-weighted and T2-weighted and Diffusive-weighted imaging (DWI) MRI [37], one used FLAIR and T2-weighted MRI [46], 2 used FLAIR and T1ce-weighted MRI [40,41], two used FLAIR, T1ce-weighted and T2-weighted MRI [45,55]. Only one paper used MRI without reporting any other information [49].

Image preprocessing techniques are essential for DL image segmentation, with considerable variability depending on the dataset employed. The included studies applied resizing to ensure consistent image dimensions [38,39,40,43,44,46,49,50,54,55,56,57,58,59,60,61,62,63,64], intensity normalization to facilitate model training [18,37,38,40,41,42,43,45,46,49,50,56,57,59,60], data augmentation to expand the dataset and enhance generalization [18,37,38,39,40,41,42,46,53,57,59,60], image cropping to manage computational load and focus on local image features [18,38,42,46,56,57,61], and noise filtering to reduce acquisition artifacts and improve the distinction of anatomical structures [38,43,54,55,61,63]. Furthermore, three studies applied postprocessing techniques to DL-segmented masks, including morphological operations [65], resampling and resizing [51], and thresholding and connected component labeling (CCL) [60].

To train and test the DL model, 22 papers used the hold-out validation approach with different split percentages [17,37,38,39,43,44,45,46,47,48,50,51,53,54,55,56,57,58,59,60,62,63]; three studies used the cross-validation approach [40,41,61]; and four papers combined the two aforementioned validation approaches [18,42,49,64]. Only one study used pre-trained models [52], and one study provided no information [65]. Furthermore, nine studies performed external validation [39,41,42,44,46,48,50,52,64].

Regarding the DL model hyperparameters, the most commonly used loss function is the Dice loss; three studies have combined it with Binary Cross-Entropy loss to improve segmentation stability and accuracy [17,39,60]. Moreover, the most used activation functions were the Rectified Linear Unit (ReLU) and sigmoid functions. The most used optimizers were the Adaptive Moment Estimation (Adam) and the Stochastic Gradient Descent (SGD). In particular, four out of 31 studies used the SGD optimizer [47,57,61,64], 15 out of 31 used the Adam optimizer [17,38,39,40,43,45,49,50,54,55,56,58,60,63,65], and two studies used both [46,53]. The learning rate used ranged from 1 × 10^−8^ [49] to 0.9 [53], and the batch size ranged from 1 to 128 [65]. Only three papers did not provide the hyperparameters used [42,52,59].

DL models were trained to segment one or several tumor regions. Furthermore, one study was trained to segment metabolic tumor volume (MTV) [42], 13 studies segmented only the WT volume [38,40,48,53,55,58,59,60,61,62,63,64,65], nine studies segmented the WT, CT, and ET [17,18,44,45,46,50,51,56,57], two studies segmented WT, ET, and NET [37,41], one study segmented NCR, ET and ED [54], and another one segmented these plus NET [43]. In addition, one paper segmented ET, NET and ED [52], one study segmented only the WT using specific datasets, while WT, CT, and ET were segmented using other datasets [39], and the remaining one segmented healthy tissue, WT, ED, active tumor, and NCR/NET regions [47]. Lastly, only one study did not report the segmented regions [49].

The specific approaches and performances of the individual studies are summarized below, highlighting the diversity of DL architecture and segmentation strategies applied across datasets and tumor subregions. Wang et al. (2022) [49] proposed a multiparametric DL framework, the Encoder–Decoder with semantic gap compensation Unit (E-DU), for brain tumor segmentation. The model is based on an encoder–decoder CNN architecture designed to address the semantic gap between low-level and high-level features. Specifically, E-DU incorporates a semantic gap compensation module that refines and aligns features extracted at different network depths, improving the consistency between encoder and decoder pathways. The proposed model achieved a DSC of 95.31%.

To assess treatment response, Gutsche et al. (2023) [42] developed a DL–based method, based on nnU-Net [74], to segment MTV. This self-configuring architecture automatically adapts preprocessing, network design, and training parameters to the dataset, ensuring data-driven performance optimization without manual tuning. The model achieved DSC values of 80.00% and 81.00%, with and without prior brain extraction, respectively. Performance was further evaluated across lesion sizes, yielding DSCs of 66.50%, 90.00%, and 87.00% for small, medium, and large lesions, respectively. Finally, nnU-Net was applied to segment single, non-malignant, and multifocal lesions, achieving DSCs of 83.00%, 77.00%, and 67.00%, respectively.

Focusing on papers that segmented only the WT, Ahsan et al. (2025) [64] used YOLOv5 for detection and a 2D U-Net for tumor segmentation. The model achieved a DSC of 88.10% for segmentation, outperforming both standalone 2D U-Net (DSC = 80.50%) and Mask R-CNN (DSC = 44.2%). Maqsood et al. (2022) [65] developed a 17-layer CNN for automatic segmentation. The CNN was designed to hierarchically extract relevant features from tumor tissue, thereby generating precise segmentation maps prior to the classification stage. This model achieved a DSC of 96.71% on BraTS2018 and of 97.87% on BTF. Furthermore, it was the only study to apply Gradient-weighted Class Activation Mapping (Grad-CAM) to explain segmentation models. The Grad-CAM analysis confirmed that the network focused on the tumor boundaries and heterogeneous internal regions, highlighting its ability to identify clinically relevant features. With the same aim of improving focus on local regions in a pre- and post-operative setting, Bouget et al. (2022) [40] developed the Attention-Gate U-Net (AGU-Net). This model extends the standard U-Net by incorporating attention gates, modules designed to highlight the most relevant regions in the input image while suppressing irrelevant background. The model demonstrated strong performance, achieving a DSC of 86.00% for HGG and 75.00% for LGG.

Following a similar approach, Rai et al. (2024) [60] employed a modified U-Net architecture with an EfficientNet encoder backbone (evaluating variants from B0 to B5) to ensure precise localization and delineation of tumor regions. The network comprises two heads: one for segmentation, and one for classification. Both heads share the EfficientNet encoder, branching only at task-specific layers, allowing the model to leverage shared features for both tasks. The segmentation performance varied across EfficientNet variants. Without postprocessing, the highest DSC was achieved by B4 and B5 (i.e., 83.50%), followed by B1 (82.56%), B3 (82.07%), B0 (80.79%), and B2 (74.22%). With postprocessing, B4 again performed best (87.00%), followed by B1 (86.62%), B5 (85.95%), B3 (84.20%), B0 (83.35%), and B2 (77.19%). In the same way, Sobhaninia et al. (2023) [61] proposed a Multiscale Cascaded Multitask Network based on a U-Net backbone with multiscale feature aggregation, designed to perform tumor segmentation and classification simultaneously. The network processes multiple resolution levels in a cascaded manner, capturing both fine and coarse features, while a feature aggregation module fuses information from different levels. The Multiple Cascaded Multitask Network reached a DSC of 95.93%, and adding feature aggregation further improved it to 96.27%, demonstrating the effectiveness of combining multiscale, cascaded, and aggregation strategies for precise tumor segmentation. Also, Roy et al. (2023) [53] proposed two lightweight U-Net–based architectures for brain tumor segmentation, i.e., S-Net and SA-Net. Both models retain the classic U-Net architecture but reduce computational overhead by using residual blocks with a single convolution per level and implementing a simplified merge strategy. SA-Net further incorporates an attention block after merging, which emphasizes salient tumor features while suppressing irrelevant regions. For S-Net, the best performance was achieved on LGG after 100 epochs, with a DSC of 81.03%, while for HGG, the peak DSC was 77.56%. For SA-Net, the highest DSC was observed on LGG after 100 epochs at 81.20%, and on HGG at 77.80%. In addition, Zafar et al. (2024) [48] introduced Enhanced TumorNet, a hybrid DL model combining YOLOv8s and U-Net for brain tumor detection and segmentation, respectively. U-Net reached an IoU of 80.00%. Furthermore, Rosa et al. (2024) [63] investigated how different image denoising techniques, such as median, Gaussian, anisotropic diffusion, and bilateral filtering, affect U-Net performance. Bilateral filtering provided the best balance between computational efficiency and segmentation accuracy, achieving a DSC of 76.96%.

Not all included studies proposed a U-Net-based model for WT segmentation. Xu et al. (2024) [62] implemented a different approach, Deep-GARL, a hybrid model that combines Generative Adversarial Networks (GANs) and Reinforcement Learning (RL) to improve segmentation of low-contrast and small brain structures. Using a semi-supervised approach, the model leverages both labeled and unlabeled data, while the RL component directs attention to relevant regions. This model reached a DSC of 85.02%. Krishnasamy et al. (2023) [38] proposed two hybrid DL models: Model 1 used a Fully Convolutional Network (FCN)-32 architecture for segmentation, followed by a ResNet-50 classifier, while Model 2 employed SegNet for segmentation with MobileNet as a classifier. The FCN-32 [75] architecture upsamples feature maps to the original image resolution, enabling accurate delineation of tumor regions, whereas SegNet [76] employs an encoder–decoder structure with pooling indices to efficiently reconstruct high-resolution segmentation maps from low-resolution feature representations. FCN-32 slightly outperformed SegNet, with DSC values of 77.40% and 76.00%, respectively. Rahat et al. (2023) [59] integrated several DL models, i.e., DeepLabv3, U-Net, DenseNet121-Unet, ResNet50, Attention U-Net, and EfficientNet, to enhance segmentation accuracy. Among these, EfficientNet achieved the highest performance with a DSC of 92.37%, followed by DenseNet121 (DSC = 85.23%) and DeepLabv1 (DSC = 84.13%).

Focusing on WT, CT, and ET segmentations, Chetty et al. (2022) [56] used a 3D U-Net, achieving DSCs of 94.00%, 90.00%, and 84.00% for WT, CT, and ET, respectively. Koteswara R.C. et al. (2022) [46] introduced two closely related architectures: Multimodal Cascaded Convolution U-Net (MCC-Net) and Multimodal Attention Cascaded U-Net (MAC-Net). MCC-Net integrates attention gates, skip connections, and group normalization to capture salient features across multiple MRI modalities, providing stability and improved performance. MAC-Net uses the same architecture without group normalization and serves as a baseline for assessing the effect of normalization. Evaluated on the BraTS 2018 dataset, MCC-Net achieved DSCs of 94.47% for WT, 84.12% for CT, and 82.72% for ET in HGG, and 85.71% for WT, 78.85% for CT, and 74.16% for ET in LGG.

Later, Mazher et al. (2024) [51] introduced the Residual Network with Multi-Head Attention (ResMHA-Net) to capture complex spatial and contextual features. ResMHA-Net achieved comparable DSC values of 90.20% for WT, 89.10% for CT, and 88.30% for ET. In the same year, Raza et al. (2023) [50], using a Residual U-Net (dResU-Net) that aimed to preserve low-level features while capturing high-level contextual information, achieved strong segmentation performance across multiple BraTS datasets. On the BraTS 2020 dataset, dResU-Net achieved DSCs of 92.12% for WT, 86.60% for CT, and 80.04% for ET. Similarly, on the BraTS 2021 dataset, it achieved DSCs of 86.01% for WT, 84.00% for CT, and 82.21% for ET.

Furthermore, Abd-Ellah et al. (2024) [57] implemented Two Parallel Cascaded U-Nets with Asymmetric Residual-blocks Network (TPCUAR-Net) to effectively capture multi-scale features by combining a symmetric U-Net architecture with asymmetric residual-blocks. The model leverages skip connections and residual units to enhance feature learning and boundary delineation, achieving DSCs of 91.00%, 83.00%, and 80.00% for WT, CT, and ET, respectively. Zaman et al. (2024) [55] used an Adaptive Feature Medical Segmentation Network (AFMS-Net). The network can incorporate two different encoder modules: the Single Adaptive Encoder Block (SAEB) for efficiency and the Dual Adaptive Encoder Block (DAEB) for complex feature extraction, along with advanced attention mechanisms to capture spatial and channel-wise dependencies. For AFMS-SAEB, the model achieved a DSC equal to 89.40%. For individual regions, DSCs were 88.00% for WT, 85.00% for CT, and 83.00% for ET. AFMS-DAEB slightly improved these results, reaching an overall DSC of 90.20%, with region-based DSCs of 87.00% for WT, 87.00% for CT, and 85.0% for ET.

Recently, Tejaswini et al. (2025) [17] improved the standard U-Net framework for automatic brain tumor segmentation by incorporating residual dense blocks, layered attention mechanisms, and class-specific attention modules. This model, called Scleral Residue Class Attention U-Net (SLCA-UNet), achieved excellent segmentation performance across multiple BraTS datasets. On the BraTS 2017 dataset, it achieved WT DSC of 91.50%, CT DSC of 85.20%, and ET DSC of 93.10%. Similar results were reported for BraTS 2018 (WT DSC = 91.50%, CT DSC = 85.20%, ET DSC = 93.10%), BraTS 2019, and BraTS 2020. Also, Aumente-Maestro C. et al. (2025) [18] introduced the Brain Tumor Segmentation (BTS) U-Net, a lightweight U-Net-based DL architecture. This model enhances the standard U-Net framework by incorporating residual dense blocks and a class-attention mechanism. For HGG, 10-fold cross-validation yielded the highest DSC of 91.10% for the WT, compared with the other subregions (89.50% for the CT and 84.80% for the ET). When trained exclusively on HGG cases, BTS U-Net further improved its performance, reaching DSCs of 91.20% for WT, 91.00% for CT, and 85.40% for ET. Yang et al. (2025) [44] introduced MUNet, which combines UNet and Mamba networks [77] for accurate brain tumor segmentation. It incorporates the Selective Scanning-State Space Model (SD-SSM) and Spatial and Channel Reconstruction Convolution (SD-Conv) modules to enhance feature extraction and computational efficiency. Evaluated on BraTS datasets, MUNet achieved DSCs of 81.50% (ET), 90.10% (WT), 81.50% (CT) on BraTS 2018, 83.50% (ET), 91.50% (WT), 82.30% (CT) on BraTS 2020, and 70.20% DSC on TCGA-LGG. Lastly, Shaikh et al. (2025) [39] investigated multiple DL models for glioma segmentation across several datasets. Compared with the other dataset used to train the model, the pretrained Visual Geometry Group (VGG)-16 [78] achieved the highest DSC of 98.03% on the Kaggle Brain Tumor MRI dataset. For the BraTS 2020 dataset, the pretrained 3D Squeeze-and-Excitation DenseNet201 (SEL-DenseNet201) obtained a DSC of 94.87% for the WT. In BraTS 2021, SEL-DenseNet201 again performed best with DSCs of 96.00% for ET, 93.02% for CT, and 93.66% for WT. Finally, on BraTS 2023, SEL-DenseNet201 reached the highest DSC values of 96.10% for ET, 93.21% for TC, and 93.78% for WT compared to the other employed datasets.

Regarding the segmentation of WT, ET, and NET, Mahajan A. et al. (2023) [37] employed a variation of 3D U-Net to segment several pediatric tumors, achieving DSCs of 61.00% for WT, 59.00% for ET, and 31.00% for NET. Improved performance in a treatment setting was reported by Zaneir et al. (2023) [41], who used a standard 2D U-Net in both pre- and post-operative segmentation settings. In the pre-operative scenario, the model achieved DSCs of 73.00% for WT, 62.00% for ET, and 43.00% for NET. In the post-operative scenario, performance decreased, with DSCs of 59.00% for WT, 21.00% for ET, and 7.00% for NET.

Esmaeilzadeh Asl et al. (2024) [54] proposed a method that uses hybrid filters to enhance the feature-extraction capabilities of the U-Net model for segmenting NCR, ED, and ET regions, achieving an overall DSC of 87.39%. Building on U-Net, Zaitoon and Sayed (2023) [43] employed 3D Residual Recurrent U-Net 2+ (3D RR-UNet2+), a 3D residual recurrent U-Net designed to capture both spatial and contextual information, to segment the same tumor subregions, while also including NET. This model demonstrated high segmentation accuracy in HGG cases, with DSCs of 98.39% for NCR, 99.10% for ED, 98.69% for ET, and 98.20% for NET. In LGG cases, performance remained excellent, with DSCs of 98.32% for NCR, 98.94% for ED, 98.94% for ET, and 99.41% for NET. In parallel, a comparative study of Kundal et al. (2024) [52] evaluated the segmentation of ET, NET, and ED using 3D CaPTk, 2DVNet, 3D EnsembleUNets, and ResNet50. CaPTk is a 3D CNN-based platform for quantitative image analysis, 2DVNet is a 2D volumetric U-Net variant, EnsembleUNets combines multiple U-Net architectures for robust segmentation, and ResNet50 is a 2D residual CNN adapted for medical imaging. On the BraTS 2021 dataset, 3D CaPTk achieved a DSC of 91.00%, 2DVNet and ResNet50 performed poorly with DSCs of 0.60%, and EnsembleUNets achieved the highest DSC of 93.00%. On the UPENN-GBM dataset, CaPTk reached a DSC of 84.00% and EnsembleUNets 85.00%, while 2DVNet and ResNet50 again showed minimal performance (1.00% DSC), highlighting the superior accuracy and robustness of 3D CaPTk and EnsembleUNets for brain tumor segmentation. Finally, Lefkovits et al. (2022) [47] employed a 3D encoder–decoder architecture for glioma segmentation, utilizing various pretrained models. Specifically, they integrated ResNet50 and ResNet101 as encoders with decoder architectures such as FCN, Pyramid Scene Parsing Network (PSPNet), and DeepLabv3+. Additionally, they implemented stacking ensemble learning (SEL) to enhance model performance. In their experiments on the Kaggle Brain Tumor MRI dataset, the best single model was VGG-16, achieving a DSC of 98.03%, while the SEL-DenseNet201 model achieved a DSC of 97.43%.

**Figure 2:**
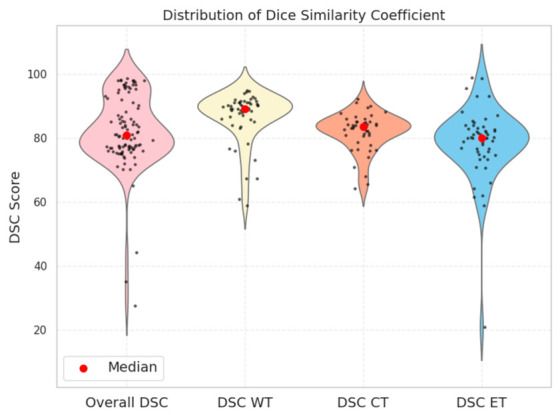
Violin plots were used to visually display the distributions of overall DSC scores and those across different tumor regions, including WT, CT, and ET. Each violin represents the distribution of all available reported DSC values. Black dots indicate individual model performance, while red dots indicate the median of each distribution. This representation allows for a clear comparison of performance variability and central tendency across tumor regions, capturing both the spread and the typical values of the reported results. Legend: DSC, Dice Score Coefficient; WT, whole tumor; CT, core tumor; ET, enhancing tumor.

To provide a clearer overview of DSC values, Figure 2 illustrates the distributions of overall DSC scores and of DSC scores across the tumor regions WT, CT, and ET. The median overall DSC was 80.73%, ranging from 0.60% to 98.59%. Considering glioma subregions, the median DSC for WT was 89.20% (range: 59.00–94.87%), for CT it was 83.56% (range: 64.26–92.30%), and for ET it was 80.04% (range: 21.00–98.94%).

**Table 1:** Overview of the included studies focusing on segmentation results.

AUT	POP	N and DB (Patients)	IMG MOD	IMG PREP	MASK POSTP	VAL APP	EV-FT DB	DL MODEL	HYPERPARAMETER	SEG AREAS	PERF	XAI
Ahsan R. et al. (2025) [64]	G, M, PT patients	233 BTF, 238 BraTS 2018	2D T1ce MRI	BTF: RZ, SS, DtL BraTS 2018: RS, DL	No	HO: 70/10/20 3-folds CV	BraTS 2018	YOLOv5 + 2D U-Net 2D U-Net Mask-RCNN: ResNet-101	YOLOv5 + 2D U-Net: lr: 0.001; opt: SGD; bs: 32; ep: 60 2D U-Net: lr: 0.001; opt: SGD, bs: 32; ep: 60 ResNet-101: lr: 0.001; opt: SGD; bs: 2; ep: 60	WT, BG	DSC	No
Maqsood S. et al. (2022) [65]	G, M, PT patients	233 BTF, 238 BraTS 2018	T1ce MRI	LCS	MOP	NA	No	17-layered CNN	lr: 0.00; Lfun: CE; Afun: ReLU, Softmax; opt: Adam; Mini-bs: 128; ep: 50	WT, BG	DSC	Grad-CAM
Ottom M.A. et al. (2022) [58]	G patients	110 TCGA-LGG	FLAIR MRI	RZ, DA	No	HO: 75/10/15	No	2D Z-Net	lr: 0.0005; Afun: ReLU, sigmoid; Lfun: BCE; opt: Adam; bs: 32; ep: 200	WT, BG	DSC, pACC, MCCoef, f1-score	No
Mahajan A. et al. (2023) [37]	EP, MD, BSG, PA patients, HC	94 CM DB	FLAIR, T1, T1ce, T2, DWI MRI	IN	No	HO: 68/10/22	No	Variation of 2D-UNet	Lfun: Dice loss; ep: 40	WT, ET, NET	DSC, HD95	No
Gutsche R. et al. (2023) [42]	G, MET, M, nMN, GG, L, EP patients	555 CM DB	18F-FET PET	BE, IC, IN	No	HO: 72/0/28 5-folds CV	33 G patients after chemotherapy	3D nnU-Net	NA	MTV, BG	V-DSC, S-DSC, k-Cohen	No
Bouget D. et al. (2022) [40]	G patients	1841 HGG, 659 LGG	T1ce, FLAIR MRI	IS, TC, SS, RZ, IN, DA	No	HGG: LOOCV LGG: 5-fold CV	No	3D AGU-Net	lr: 0.001; Lfun: Dice loss; Bs: 32; opt: Adam; es: patience = 30	WT, BG	Voxel-wise: DSC, Dice-TP Patient- and Object-wise: f1-score, SN, PC	No
Abd-Ellah M.K. et al. (2024) [57]	G patients	170 BraTS 2017	3D FLAIR, T1, T1ce, T2 MRI	IC, IN, RZ, DA	No	HO: 60/0/40	No	TPCUAR-Net	lr: 0.08; Afun: PReLU, Softmax; opt: SGD; Mom: 0.9; bs: 16; ep: 100	WT, CT, ET	DSC	No
Zanier O. et al. (2023) [41]	G patients	1455 BraTS 2021, CM DB Zurich (preoperative, post-operative), BraTS 2015	T1ce, FLAIR MRI	RTT, SS, BM, IN, DA	No	5-folds CV	CM DB Zurich post-operative	2D UNet	lr: 0.001; opt: Ranger PCE; bs 32; ep: 40 (pre-operative), 15 (post-operative)	WT, ET, NET	DSC, IoU, HD95	No
Rai H.M. et al. (2024) [60]	G, M, PT patients	233 BTF	2D T1ce MRI	RZ, IN, DA	CCL, Thresholding	HO: 87/12/1	No	2D Two-headed UNetEfficientNets	UNetEfficientNets-B0: lr: 0.01 (encoder), 0.001 (decoder); Lfun: 70% BCE + 30% Dice loss; Bs: 16; opt (decoder): AdamW UNetEfficientNets-B1: lr: 0.01 (encoder), 0.001 (decoder); Lfun: 70% BCE + 30% Dice loss; Bs: 12; opt (decoder): AdamW UNetEfficientNets-B2: lr: 0.01 (encoder), 0.001 (decoder); Lfun: 70% BCE + 30% Dice loss; Bs: 12; opt (decoder): AdamW UNetEfficientNets-B3: lr: 0.01 (encoder), 0.001 (decoder); Lfun: 70% BCE + 30% Dice loss; Bs: 10; opt (decoder): AdamW UNetEfficientNets-B4: lr: 0.01 (encoder), 0.001 (decoder); Lfun: 70% BCE + 30% Dice loss; Bs: 4; opt (decoder): AdamW UNetEfficientNets-B5:lr: 0.01 (encoder), 0.001 (decoder); Lfun: 70% BCE + 30% Dice loss; Bs: 2; opt (decoder): AdamW	WT, BG	DSC	No
Chetty G. et al. (2022) [56]	G patients	65 LGG BraTS 2018	FLAIR, T1, T1ce, T2 MRI	IC, IN, RZ	No	HO: 60/20/20	No	3D U-Net	lr: 0.0001; Afun: ReLU, sigmoid; Lfun: Dice loss; opt: Adam; bs: 64; ep: 30	WT, CT, ET	DSC	No
Tejashwini P.S. et al. (2025) [17]	G patients	285 BraTS 2017, 238 BraTS 2018, 335 BraTS 2019, NA BraTS 2020	FLAIR, T1, T1ce, T2 MRI	No	No	HO: 70/0/30, 80/0/20	No	SLCA U-Net	lr: 0.001; Afun: ReLU; Lfun: CE, Dice loss; bs: 16; ep: 50; opt: Adam; dp: 0.5	WT, CT, ET	pACC, SN, SP, DSC and HD95	No
Zaman A. et al. (2024) [55]	G patients	1251 BraTS 2021	3D FLAIR, T1ce, T2 MRI	NF, RZ	No	HO: 80/20/0	No	3D AFMS-SAEB, AFMS-DAEB models	lr: 0.0001; Lfun: Dice loss, categorical focal loss; opt: Adam; wd: 0.0005	NCR, ED, ET, NET	pACC, SN, PC, DSC, IoU, HD95	No
Sobhaninia Z. et al. (2023) [61]	G, M, PT patients	233 BTF	T1ce MRI	MF, CLAHE, IC	No	5-folds CV	No	Multiscale Cascaded Multitask network	lr: 0.001; Lfun: Dice loss; opt: SGD; ep: 150	WT, BG	DSC, IoU	No
Esmaeilzadeh Asl S. et al. (2024) [54]	G patients	369 BraTS 2020	FLAIR, T1, T1ce, T2 MRI	BF, MOP, RZ	No	HO: 90/10/0	No	2D U-Net	Lfun: Dice loss; Afun: ReLU; opt: Adam; ep: 50; bs: 85	NCR, ED, ET	SN, SP, DSC, HD95	No
Roy et al. (2023) [53]	G patients	75 LGG: BraTS 2018, BraTS 2019, BraTS 2020 200 HGG: BraTS 2018, BraTS 2019, BraTS 2020, BraTS 2021	FLAIR, T1, T1ce, T2 MRI	2D Tr, DA	No	HO: 80/10/10	No	2D S-Net, 2D SA-Net	lr: 0.001, 0.9, 0.0005; opt: SGD, Adam; Afun: ReLU Lfun: BCE; dp: 0.2, 0.5, 0.2 + 0.5; ep: 20, 50, 75, 100; bs: 16, 32, 64	WT, BG	IoU, DSC, SN, SP, pACC	No
Aumente-Maestro C. et al. (2025) [18]	G patients	369 BraTS 2020	FLAIR, T1, T1ce, T2 MRI	VOR, IN, IC, DA	No	HO: 80/10/10 10-folds CV	No	3D BTS U-Net	lr: 0.001–0.000001; opt: Ranger; bs: 1; ep: 400; Es: patience = 20	WT, CT, ET	DSC	No
Kundal K. et al. (2024) [52]	G patients	1251 BraTS 2021, 611 UPENN-GBM	FLAIR, T1, T1ce, T2 MRI	No	No	Pre-trained models	BraTS 2021, UPENN-GBM dataset	3D CaPTk, 2DVNet, 3D EnsembleUNets, ResNet50	NA	ET, NET, ED	DSC, HD95	No
Xu C. et al. (2024) [62]	G, M, PT patients	233 BTF	T1ce MRI	RZ	No	HO: 80/0/20, 20/0/80, 60/0/40, 40/0/60	No	DGARL	lr: 0.0001	WT, BG	DSC, HD95, IoU, SN, SP, k-Cohen	No
Krishnasamy N. et al. (2023) [38]	G, M, PT patients, HC	3264 K-BT	T1ce MRI	IC, MOP, RZ, IN, DA	No	HO: 80/0/20, 90/0/10	No	FCN-32, SegNet	lr: 0.0001; lr decay: 0.9; ep: 250; opt: Adam; Afun: ReLU; Test bs: 20	WT, BG	DSC, IoU	No
Mazher M. et al. (2024) [51]	G patients	369 BraTS 2020	FLAIR, T1, T1ce, T2 MRI	No	RS, RZ	HO: 80/0/20	No	3D CNN model + 3D HAM + MSAFE module	lr: 0.0001; Afun: ReLU, sigmoid; Lfun: Combo function	WT, CT, ET	DSC, HD95	No
Raza R. et al. (2023) [50]	G patients	369 BraTS 2020, 50 BraTS 2021	FLAIR, T1, T1ce, T2 MRI	IN, RZ, IMS	No	HO: 80/10/10	BraTS 2021	3D dResU-Net	lr: 0.0001; Afun: ReLU, Softmax; Lfun: focal loss, soft dice loss; opt: Adam; ep: 100; bs: 4; dp: 0.1	WT, CT, ET	DSC, SN, SP	No
Wang H. et al. (2022) [49]	G patients	274 BraTS 2015	MRI	IN, RZ, RM	No	HO: 80/0/20 5-folds CV	No	2D E-DU net	lr: 1 × 10^−8^; Afun: ReLU; opt: Adam; ep: 300	NA	DSC, IoU, MCCoef, SN, PC, f2-score	No
Shaikh A. et al. (2025) [39]	G, M, PT patients, HC	NA K-BT, NA K-BTC, 369 BraTS 2020, NA BraTS 2021, NA BraTS 2023	3D FLAIR, T1, T1ce, T2 MRI	RZ, DA	No	HO: 70/10/20	K-BT, K-BTC, BraTS 2020, BraTS 2021, BraTS 2023	(all pretrained) 3D-CNN, ResNet50, MobileNet-v3, VGG-16, VGG 19, AlexNet, 3D SEL-DenseNet201	lr: 0.0001; Lfun: CE, Dice loss; opt: Adam; Bs: 1; ep: 25, dp: NA	K-BT, K-BTC: WT, BG BraTS2020, BraTS2021, BraTS2023: WT, CT, ET	pACC, SN, SP, PC, f1-score, MCCoef, AUC, DSC	No
Zafar W. et al. (2024) [48]	G patients	110 TCGA-LGG	FLAIR MRI	No	No	HO	TCGA-LGG	pretrained YOLOv8s + U-Net	lr: 0.0001; Lfun: CE ep: 100; Bs: 16–32	WT, BG	pACC, PC, SN, SP, f1-score, AUC, IoU	No
Rosa S. et al. (2024) [63]	G, M, PT patients	233 BTF	T1ce MRI	RZ, MF, GF, AF, BF	No	HO: 60/20/20	No	U-Net	lr: 1 × 10^−4^–1 × 10^−7^; Afun: ReLU, sigmoid; Lfun: Dice loss; opt: Adam; ep: 300; bs: 115; es: patience = 20	WT, BG	pACC, SN, PC, DSC, IoU	No
Lefkovits S. et al. (2022) [47]	Glioma patients	NA BraTS 2017, BraTS 2018, BraTS 2019, BraTS 2020	3D FLAIR, T1, T1ce, T2 MRI	NA	No	HO: 60/20/20	No	3D Encoder (pretrained ResNet50/ResNet101)-decoder (FCN, PSPNet, and DeepLabv3+) based network Ensemble	lr: 0.001; Lfun: Jaccard loss; ep: 100; opt: SGD; Mom: 0.9; es: patience = 4 FSC-ResNet50 hyperparameter optimization: lr: 0.0009; wd: 0.0114; Mom: 0.803; opt: AdaGrad	BG, HT, WT, ED, AT, NCR/NET	DSC	No
Koteswara R.C. et al. (2022) [46]	G patients	285 BraTS 2018, 335 BraTS 2019, 369 BraTS 2020	FLAIR, T2 MRI	IN, IC, RZ, DA	No	HO: 80/0/20	BraTS 2019, BraTS 2020	MCC U-Net + BN, MCC U-Net + GN, MAC U-Net + BN, MAC U-Net + GN	lr: 0.0001; Lfun: Dice loss; Afun: ReLU; bs: 8; ep: 30; opt: Adam, SGD, RMSprop	WT, CT, ET	pACC, DSC, IoU, SN, SP	No
Sachdeva J. et al. (2024) [45]	G patients	369 BraTS 2020, 1254 BraTS 2021, 92 PGIMER	3D FLAIR, T1ce, T2 MRI	SS, BC, IN, SR, RZ, DR	No	HO: 75/0/25	No	3D MS-SegNet	lr: 0.0001; Afun: ReLU; Lfun: Dice loss, focal loss; opt: Adam; ep: 100; bs: 2	WT, CT, ET	DSC, SP, SN, HD95	No
Yang L. et al. (2025) [44]	G patients	285 BraTS 2018, 369 BraTS 2020, NA TCGA-LGG	3D FLAIR, T1, T1ce, T2 MRI	No	No	HO	TCGA-LGG	M U-Net	Lfun: combination of mIoU, Dice loss, Boundary loss	WT, CT, ET	k-Cohen, DSC, IoU, SP, SN, PC, pACC, bACC, HD95, AUC	No
Zaitoon R. et al. (2023) [43]	G patients	285 BraTS 2017, 266 BraTS 2018, 285 BraTS 2019, 335 BraTS 2020	3D FLAIR, T1, T1ce, T2 MRI	MF, RZ, IN	No	HO: 70/10/20	No	3D RRUNet2+	lr: 0.001; Lfun: BCE; Afun: ReLU; opt: Adam; dp: 0.1; ep: 10; bs: 32	NCR, ED, ET, NET	DSC, pACC	No
Rahat I.S. et al. (2023) [59]	G patients	150 TCGA-LGG	FLAIR MRI	RZ, IN, DA	No	HO: 70/15/15	No	3D U-Net	NA	WT, BG	DSC, IoU, TI	No

Legend: AUT: Authors, POP: Population, N: Numerosity, DB: Dataset, IMG: image, MOD: Modality, PREP: Preprocessing, POSTP: Postprocessing, VAL APP: Validation approach, EV-FT DB: External Validation/Fine tuning dataset, DL: Deep Learning, SEG: segmented, PERF: performance, XAI: Explainable Artificial Intelligence, NA: Not Available, G: glioma, M: meningioma, PT: pituatory tumor, EP: Ependymoma, MD: medulloblastoma, BSG: brainstem glioma, PA: pilocytic astrocytoma, MET: metastasis, nMN: non-malignant neoplasm, GG: ganglioma, L: lymphoma, CM: custom-made, HT: Healthy Tissue, AT: Active tumor, BG: Background, WT: whole Tumor, NCR: Necrotic Tumor Region, NET: Non-enhancing tumor regions, ED: Edematous tissue, ET: Enhancing tumor regions, CT: Core Tumor, DSC: Dice Score Coefficient, MTV: Metabolic tumor volume, LGG: Low grade glioma, HGG: High grade glioma, T1ce: contrast-enhanced, BTF: Brain Tumor Figshare, K-BT: Kaggle Brain Tumor dataset, K-BTC: Kaggle Brain Tumor classification dataset, TCGA-LGG: The Cancer Genome Atlas Low-Grade Glioma, HO: Hold-out, CV: cross-validation, LOOCV: leave-one-out cross-validation, RZ: resize, SS: skull-stripping, DtL: data labeling, LCS: linear contrast stretching, DA: data augmentation, IN: intensity normalization, BE: brain extraction, IC: image cropping, TC: tight cropping, IS: isotropic spacing, RTT: rigid transformation technique, BM: brain masking, NF: noise filtering, MF: median filter, VOR: voxel outliers removing, SR: spatial registration, RS: resampling, BC: bias-correction, GF: gaussian filter, AF: anisotropic filter, BF: bilateral filter, IMS: image stacking, RM: resolution modification, DR: lossless dimensionality reduction, MOP: morphological operations, lr: learning rate, opt: optimizer, bs: batch size, ep: epochs, Lfun: loss function, Afun: activation function, dp: drop-out rate, wd: weight decay, Mom: momentum, es: early stopping, HD95: 95th percentile Hausdorff Distance, pACC: pixel accuracy, MCCoef: Matthews Correlation Coefficient, AUC: Area Under the Curve, V-DSC: Volumetric-DSC, S-DSC: Surface-DSC, IoU: Intersection over Union, AFMS-SAEB: Adaptive Feature Medical Segmentation-Single Adaptive Encoder Block, AFMS-DAEB: Adaptive Feature Medical Segmentation-Dual Adaptive Encoder Block, CLAHE: Contrast limited histogram equalization, BTS: Brain Tumor Segmentation, SGD: Stochastic Gradient Descent, BCE: binary cross-entropy loss, CE: cross-entropy loss, HAM: hierarchical attention module, MSAFE: multi-scale-aware feature enhancement, dResU-Net: Deep Residual U-Net, SEL: stacking ensemble learning, VGG: visual geometry group, k: kernel size, K: gradient sensibility, n: number of iterations, FCN: Fully Convolutional Network, PSPNet: Pyramid Scene Parsing Network, MAC: Multimodal attention-gated cascaded, MCC: Multi-Channel cascaded, BN: Batch Normalization, GN: Group Normalization, MS-SegNet: multi-scale segmentation architecture, PGIMER: Post Graduate Institute of Medical Education & Research, Chandigarh, SP: specificity, SN: sensitivity, PC: Precision, bACC: Balanced Accuracy, RRUNet2+: Recurrent Residual U-Net 2+, TPCUAR-Net: Two parallel cascaded U-Nets with an asymmetric residual, TI: Tversky index, DGARL: Deep Generative Adversarial Reinforcement Learning, AGU-Net: Attention Gate U-Net, CCL: connected component labeling.

**Table 2:** Summary of the major findings of the studies included in the review.

AUTHORS	MAJOR FINDINGS
Ahsan R. et al. (2025) [64]	YOLOv5 + 2D U-Net: DSC = 88.10%; 2D U-Net: DSC = 80.50%; Mask R-CNN: DSC = 44.2%
Maqsood S. et al. (2022) [65]	**BraTS 2018:** DSC = 96.71%; **BTF:** DSC = 97.87%
Ottom M.A. et al. (2022) [58]	U-Net: DSC = 85.00%, pACC = 99.00%; f1-score = 79.00%; MCCoef = 78.00%; Z-Net: DSC = 92.00%, pACC = 100.00%; f1-score = 81.00%; MCCoef = 81.00%
Mahajan A. et al. (2023) [37]	WT: DSC = 61.00%, HD95 = 8.10; ET: DSC = 59.00%, HD95 = 3.15; NET: DSC = 31.00%, HD95 = 21.26
Gutsche R. et al. (2023) [42]	No prior brain extraction: V-DSC = 81.00%, S-DSC = 96.00%; With prior brain-extraction: V-DSC = 80%, S-DSC = 95.00%; small lesions: V-DSC = 66.50%, S-DSC: 92%; medium lesions: V-DSC = 80.00%, S-DSC = 93.00%; large lesions: V-DSC = 87.00%, S-DSC = 97.00%; single lesion: V-DSC = 83.00%, S-DSC = 96.00%; Nonmalignant: V-DSC = 77.00%, S-DSC = 93.00%; Multi-focal: V-DSC = 67.00%, S-DSC = 81.00%; k-Cohen baseline = 0.81; k-Cohen follow-up = 0.95
Bouget D. et al. (2022) [40]	**LGG:** DSC = 75.00%, Dice-TP = 81.00%; Patient-wise: f1-score = 94.00%, SN = 93.00%, PC = 94.00%; Object-wise: f1-score = 84.00%, SN = 84.00%, PC = 81.00%; **HGG:** DSC = 86.00%, Dice-TP = 87.00%; Patient-wise: f1-score = 97.00%, SN = 98.00%, PC = 97.00%; Object-wise: f1-score = 90.00%, SN = 86.00%, PC = 94.00%
Abd-Ellah M.K. et al. (2024) [57]	WT: DSC = 91.00%; CT: DSC = 83.00%; ET: DSC = 80.00%
Zanier O. et al. (2023) [41]	**Pre-operative:** ET: DSC = 62.00%, IoU = 56.00%, HD95 = 5.30; WT: DSC = 73.00%, IoU = 57.00%, HD95 = 8.07; NET: DSC = 43.00%, IoU = 38.00%, HD95 = 10.26 **Post-operative:** ET: DSC = 21.00%, IoU = 17.00%, HD95 = 13.18; WT: DSC = 59.00%, IoU = 50.00%, HD95 = 14.08; NET: DSC = 7.00%, IoU = 5.00%, HD95 = 20.16
Rai H.M. et al. (2024) [60]	**Without postp:** UNetEfficientNet-B0: DSC = 80.79%; UNetEfficientNet-B1: DSC = 82.56%; UNetEfficientNet-B2: DSC = 74.22%; UNetEfficientNet-B3: DSC = 82.07%; UNetEfficientNet-B4: DSC = 83.50%; UNetEfficientNet-B5: DSC = 83.50%; **With postp:** UNetEfficientNet-B0: DSC = 83.35%; UNetEfficientNet-B1: DSC = 86.62%; UNetEfficientNet-B2: DSC = 77.19%; UNetEfficientNet-B3: DSC = 84.20%; UNetEfficientNet-B4: DSC = 87.00%; UNetEfficientNet-B5: DSC = 85.95%
Chetty G. et al. (2022) [56]	ET: DSC = 84%; WT: DSC = 94%; CT: DSC = 90%
Tejashwini P.S. et al. (2025) [17]	**BraTS 2017 70/30:** pACC = 98.95; **80/20:** pACC = 99.96%; **BraTS 2017:** WT: DSC = 91.50%, HD95 = 3.80; CT: DSC = 85.20%, HD95 = 5.10; ET: DSC = 93.10%, HD95 = 3.55; **BraTS 2018:** WT: DSC: 91.50%, HD95 = 3.80; CT: DSC = 85.20%, HD95 = 5.10; ET: DSC = 93.10%, HD95 = 3.55 **BraTS 2019:** WT: DSC = 89.00%, SP = 99.00%, SN = 94.00%, HD95 = 7.30; CT: DSC = 86.00%, SP = 99.00%, SN = 89.00%, HD95 = 8.10; ET: DSC = 84.00%, SP = 99.00%, SN = 88.00%, HD95 = 24.50; **BraTS 2020:** WT: DSC = 90.00%, SP = 99.00%, SN = 94.00%, HD95 = 7.30; CT: DSC = 84.00%, SP = 99.00%, SN = 84.00%, HD95 = 8.10; ET: DSC = 82.00%, SP = 99.00%, SN = 83.00%, HD95 = 24.50
Zaman A. et al. (2024) [55]	**AFMS-SAEB:** Overall segmentation: pACC = 98.9%, PC = 91.3%, SN = 88.5%, DSC = 89.4%, IoU = 80.6%, HD95 = 6.27; WT: pACC = 98.0%, PC = 91.0%, SN = 85.0%, DSC = 88.0%, IoU = 79.0%; CT: pACC = 98.0%, PC = 86.0%, SN = 85.0%, DSC = 85.0%, IoU = 75.0%; ET: pACC = 98.0%, PC = 87.0%, SN = 78.0%, DSC = 83.0%, IoU = 71.0%; **AFMS-DAEB:** Overall segmentation: pACC = 99.0%, PC = 90.8%, SN = 89.6%, DSC = 90.2%, IoU = 81.3%, HD95 = 6.08; WT: pACC = 99.0%, PC = 84.0%, SN = 91.0%, DSC = 87.0%, IoU = 78.0%; CT: pACC = 99.0%, PC = 88.0%, SN = 86.0%, DSC = 87.0%, IoU = 77.0%; ET: pACC = 99.0%, PC = 87.0%, SN = 83.0%, DSC = 85.0%, IoU = 74.0%
Sobhaninia Z. et al. (2023) [61]	**Multiscale Cascade Network Without Detection:** DSC = 84.73%, IoU = 82.54%; **Multiscale Cascade Network With Detection:** DSC = 94.35%, IoU = 92.09%; **Multiple Cascaded Multitask Network:** DSC = 95.93%, IoU = 94.75%; **Multiple Cascaded Multitask Network with Aggregation:** DSC = 96.27%, IoU = 95.88%
Esmaeilzadeh Asl S. et al. (2024) [54]	SN = 97.36%, SP = 99.63%, DSC = 87.39%, HD95 = 5.93
Roy et al. (2023) [53]	**HGG S-Net ep:** ep = 20: IoU: 76.63%, DSC: 76.84%, SN: 96.91%, SP: 96.94%, pACC: 93.32%; ep = 50: IoU: 77.20%, DSC: 77.40%, SN: 95.89%, SP: 97.96%, pACC: 94.65%; ep = 75: IoU: 77.30%, DSC: 77.50%, SN: 95.61%, SP: 98.19%, pACC: 94.51%; ep = 100: IoU: 77.36%, DSC: 77.56%, SN: 95.31%, SP: 98.34%, pACC: 94.65%; **HGG S-Net dp:** dp = 0.2: IoU: 76.63%, DSC: 76.84%, SN: 96.91%, SP: 96.94%, pACC: 93.32%; dp = 0.5: IoU: 75.78%, DSC: 76.84%, SN: 97.57%, SP: 95.60%, pACC: 92.05%; dp = 0.2 + 0.5: IoU: 76.25%, DSC: 76.46%, SN: 97.91%, SP: 96.23%, pACC: 92.66%; **LGG S-Net ep**: ep = 20: IoU: 79.76%, DSC: 79.93%, SN: 95.08%, SP: 97.15%, pACC: 93.64%; ep = 50: IoU: 80.75%, DSC: 80.91%, SN: 94.46%, SP: 98.55%, pACC: 94.99%; ep = 75: IoU: 80.85%, DSC: 81.01%, SN: 93.85%, SP: 98.72%, pACC: 95.15%; ep = 100: IoU: 80.87%, DSC: 81.03%, SN: 92.80%, SP: 98.78%, pACC: 95.21%; **LGG S-Net dp:** dp = 0.2: IoU: 79.76%, DSC: 79.93%, SN: 95.08%, SP: 97.15%, pACC: 93.64%; dp = 0.5: IoU: 77.87%, DSC: 78.05%, SN: 96.84%, SP: 94.1%, pACC: 91.00%; dp = 0.2 + 0.5: IoU: 75.51%, DSC: 75.70%, SN: 97.66%, SP: 90.69%, pACC: 87.45%; **HGG SA-Net ep**: ep = 20: IoU: 77.20%, DSC: 77.39%, SN: 95.26%, SP: 98.14%, pACC: 94.46%; ep = 50: IoU: 77.54%, DSC: 77.72%, SN: 94.34%, SP: 98.78%, pACC: 95.06%; ep = 75: IoU: 77.59%, DSC: 77.79%, SN: 93.53%, SP: 99.00%, pACC: 95.27%; ep = 100: IoU: 77.61%, DSC: 77.80%, SN: 93.33%, SP: 99.03%, pACC: 95.29%; **HGG SA-Net dp**: dp = 0.2: IoU: 77.20%, DSC: 77.39%, SN: 95.26%, SP: 98.14%, pACC: 94.46%; dp = 0.5: IoU: 77.06%, DSC: 77.39%, SN: 96.27%, SP: 97.80%, pACC: 94.15%; dp = 0.2 + 0.5: IoU: 76.35%, DSC: 76.55%, SN: 97.47%, SP: 96.54%, pACC: 92.95%; **LGG SA-Net ep**: ep = 20: IoU: 79.90%, DSC: 80.08%, SN: 96.40%, SP: 97.16%, pACC: 93.66%; ep = 50: IoU: 80.96%, DSC: 81.12%, SN: 92.89%, SP: 98.98%, pACC: 95.39%; ep = 75: IoU: 81.03%, DSC: 81.18%, SN: 91.78%, SP: 99.17%, pACC: 95.64%; ep = 100: IoU: 81.05%, DSC: 81.20%, SN: 91.41%, SP: 99.25%, pACC: 95.64%; **LGG SA-Net dp:** dp = 0.2: IoU: 79.90%, DSC: 80.08%, SN: 96.40%, SP: 97.16%, pACC: 93.66%; dp = 0.5: IoU: 79.38%, DSC: 79.56%, SN: 95.91%, SP: 96.59%, pACC: 93.12%; dp = 0.2 + 0.5: IoU: 77.83%, DSC: 78.02%, SN: 98.10%, SP: 93.96%, pACC: 90.61%
Aumente-Maestro C. et al. (2025) [18]	**BTS U-Net HO:** ET: DSC = 81.10%; CT: DSC = 87.80%; WT: 90.80%; **BTS U-Net 10-folds CV:** ET: DSC = 80.00%; CT: DSC = 85.70%; WT: DSC = 90.70%; ET HGG: DSC = 84.80; CT HGG: DSC = 89.50%; WT HGG: DSC = 91.10%; ET LGG: LGG DSC: 61.60%; CT LGG: DSC = 70.80%; WT LGG: DSC = 89.00%; **BTS U-Net 10-folds CV on HGG:** ET: DSC = 85.40%; CT: DSC = 91.00%; WT: DSC = 91.20%
Kundal K. et al. (2024) [52]	**BraTS 2021:** CaPTk: DSC = 91.00%, HD95 = 32.95; 2DVNet: DSC = 0.60%, HD95 = 105.2; EnsembleUNets: DSC = 93.00%, HD95 = 18.14; ResNet50: DSC = 0.60%, HD95 = 110; **UPENN-GBM:** CaPTk: DSC = 84.00%, HD95 = 29.04; 2DVNet: DSC = 1.00%, HD95 = 106.95; EnsembleUNets: DSC = 85.00%, HD95 = 17.46; ResNet50: DSC = 1.00%, HD95 = 113.66
Xu C. et al. (2024) [62]	80/0/20: DSC = 85.02%, HD95 = 4.64, IoU = 77.51%, k-Cohen = 84.75%, SN = 87.72%, SP = 99.72%; 20/0/80: DSC = 71.80%; 60/0/40: DSC = 79.36%; 40/0/60: 75.97%
Krishnasamy N. et al. (2023) [38]	FCN-32: DSC = 77.40%, IoU = 75.00%; SegNet: DSC = 76.00%, IoU = 74.00%
Mazher M. et al. (2024) [51]	ET: DSC = 88.30%, HD95 = 4.18; CT: DSC = 89.10%, HD95 = 3.62; WT: DSC = 90.20%, HD95 = 2.89
Raza R. et al. (2023) [50]	**BraTS 2020:** WT: DSC = 92.12%, SN = 98.68%, SP = 98.64%; CT: DSC = 86.60%, SN = 97.86%, SP = 99.36%; ET: DSC = 80.04%, SN = 96.48%, SP = 97.91%; **BraTS 2021:** WT: DSC = 86.01%, SN = 98.17%, SP = 99.45%; CT: DSC = 84.00%, SN = 97.81%, SP = 98.36%; ET: DSC = 82.21%, SN = 97.13%, SP = 97.45%
Wang H. et al. (2022) [49]	DSC = 95.31%, f2-score = 93.50%, IoU = 85.71%, SN = 93.67%, PC = 92.85%, MCCoef = 92.38%
Shaikh A. et al. (2025) [39]	**K-BT:** 3D-CNN: pACC = 96.63%, SP = 95.76%, SN = 95.87%, PC = 96.78%, f1-score = 96.43%, MCCoef = 96.60%, DSC = 96.60%, AUC = 98.00%; VGG-19: pACC = 97.00%, SP = 96.65%, SN = 97.15%, PC = 97.20%, f1-score = 97.61%, MCCoef = 97.51%, DSC = 97.51%, AUC = 97.00%; VGG-16: pACC = 98.60%, SP = 97.33%, SN = 97.62%, PC = 97.42%, f1-score = 97.72%, MCCoef = 98.03%, DSC = 98.03%, AUC = 94.00%; MobileNet-V3: pACC = 96.00%, SP = 96.34%, SN = 96.55%, PC = 96.30%, f1-score = 95.60%, MCCoef = 96.62%, DSC = 96.62%, AUC = 99.00%; AlexNet: pACC = 96.56%, SP = 96.70%, SN = 95.65%, PC = 96.44%, f1-score = 95.82%, MCCoef = 95.60%, DSC = 95.60%, AUC = 98.00%; ResNet50: pACC = 95.49%, SP = 95.37%, SN = 95.29%, PC = 95.37%, f1-score = 95.57%, MCCoef = 95.48%, DSC = 95.48%, AUC = 99.00%; SEL-DenseNet201: pACC = 99.65%, SP = 100.00%, SN = 98.97%, PC = 99.76%, f1-score = 98.99%, MCCoef = 99.87%, DSC = 97.43%, AUC = 100.00% **K-BTC:** 3D-CNN: pACC = 97.40%, SP = 96.60%, SN = 96.40%, PC = 97.80%, f1-score = 96.43%, MCC = 97.60%, AUC = 98.00%**;** VGG-19: pACC = 97.30%, SP = 97.50%, SN = 97.30%, PC = 96.40%, f1-score = 97.61%, MCC = 96.51%, AUC = 97.00%; VGG-16: pACC = 98.00%, SP = 96.73%, SN = 96.62%, PC = 96.50%, f1-score = 97.72%, MCC = 96.33%, AUC = 93.00%; MobileNet-V3: pACC = 96.80%, SP = 97.40%, SN = 96.05%, PC = 97.00%, f1-score = 95.60%, MCC = 97.02%, AUC = 95.00%; AlexNet: pACC = 96.60%, SP = 96.65%, SN = 96.71%, PC = 97.40%, f1-score = 95.82%, MCC = 96.00%, AUC = 97.00%; ResNet50: pACC = 96.50%, SP = 95.37%, SN = 95.90%, PC = 96.70%, f1-score = 95.57%, MCC = 96.08%, AUC = 97.00%; SEL-DenseNet201: pACC = 98.65%, SP = 98.60%, SN = 97.43%, PC = 98.74%, f1-score = 97.66%, MCC = 97.22%, DSC = 96.61, AUC = 98.00%; **BraTS 2020:** 3D-CNN: pACC = 95.10%, SP = 96.50%, SN = 97.20%, PC = 96.80%, f1-score = 97.00%, MCC = 96.00%, AUC = 98.00%; VGG-19: pACC = 96.00%, SP = 96.50%, SN = 96.50%, PC = 97.70%, f1-score = 96.50%, MCC = 96.30%, AUC = 97.00%; VGG-16: pACC = 96.30%, SP = 96.30%, SN = 96.30%, PC = 97.20%, f1-score = 97.20%, MCC = 96.10%, AUC = 93.00%; MobileNet-V3: pACC = 97.40%, SP = 96.40%, SN = 97.50%, PC = 96.10%, f1-score = 97.30%, MCC = 96.80%, AUC = 98.00%; AlexNet: pACC = 96.20%, SP = 96.50%, SN = 97.10%, PC = 96.80%, f1-score = 96.80%, MCC = 96.50%, AUC = 98.00%; ResNet50: pACC = 96.60%, SP = 97.60%, SN = 96.90%, PC = 97.10%, f1-score = 97.40%, MCC = 97.40%, AUC = 97.00%; SEL-DenseNet201: pACC = 98.98%, SP = 98.90%, SN = 98.73%, PC = 97.99%, f1-score = 99.00%, MCC = 97.02%, DSC = 94.24%, AUC = 99.00%; ET: DSC = 95.56%, HD = 3.01; TC: DSC = 92.30%, HD = 4.33; WT: DSC = 94.87%, HD = 4.60; **BraTS 2021**: 3D-CNN: pACC = 97.20%, SP = 97.50%, SN = 97.60%, PC = 98.00%, f1-score = 97.00%, MCC = 97.00%, AUC = 98.00%; VGG-19: pACC = 98.20%, SP = 97.50%, SN = 97.00%, PC = 97.00%, f1-score = 96.70%, MCC = 96.10%, AUC = 96.00%; VGG-16: pACC = 98.20%, SP = 96.30%, SN = 96.80%, PC = 96.80%, f1-score = 96.80%, MCC = 96.30%, AUC = 93.00%; MobileNet-V3: pACC = 97.50%, SP = 96.40%, SN = 96.80%, PC = 97.00%, f1-score = 96.90%, MCC = 97.20%, AUC = 98.00%; AlexNet: pACC = 97.60%, SP = 96.50%, SN = 97.50%, PC = 96.90%, f1-score = 97.00%, MCC = 95.60%, AUC = 98.00%; ResNet50: pACC = 97.50%, SP = 96.30%, SN = 96.97%, PC = 96.80%, f1-score = 96.30%, MCC = 96.80%, AUC = 98.00%; SEL-DenseNet201: pACC = 99.00%, SP = 98.50%, SN = 98.93%, PC = 98.98%, f1-score = 98.60%, MCC = 97.30%, DSC = 94.23%, AUC = 100.00%; ET: DSC = 96.00%, HD = 3.00; TC: DSC = 93.02%, HD = 4.56; WT: DSC = 93.66%, HD = 3.75; **BraTS 2023:** 3D-CNN: pACC = 98.00%, SP = 97.40%, SN = 97.30%, PC = 97.30%, f1-score = 97.10%, MCC = 97.00%, AUC = 98.00%; VGG-19: pACC = 97.30%, SP = 97.30%, SN = 97.10%, PC = 97.60%, f1-score = 97.80%, MCC = 96.00%, AUC = 97.00%; VGG-16: pACC = 97.80%, SP = 97.60%, SN = 97.70%, PC = 96.90%, f1-score = 97.30%, MCC = 96.10%, AUC = 93.00%; MobileNet-V3: pACC = 96.70%, SP = 96.30%, SN = 96.10%, PC = 96.40%, f1-score = 96.00%, MCC = 96.20%, AUC = 96.00%; AlexNet: pACC = 97.20%, SP = 96.80%, SN = 96.70%, PC = 96.10%, f1-score = 96.00%, MCC = 95.40%, AUC = 96.00%; ResNet50: pACC = 96.50%, SP = 96.70%, SN = 95.90%, PC = 96.60%, f1-score = 96.80%, MCC = 96.30%, AUC = 98.00%; SEL-DenseNet201: pACC = 98.10%, SP = 98.30%, SN = 98.20%, PC = 98.80%, f1-score = 98.00%, MCC = 98.40%, DSC = 94.36%, AUC = 98.00%; ET: DSC = 96.10%, HD = 3.02; TC: DSC = 93.21%, HD = 4.21; WT: DSC = 93.78%, HD = 4.05
Zafar W. et al. (2024) [48]	pACC = 98.60%, SN = 97.80%, recall = 95.2%, f1-score = 96.30%, SP = 89.1%, AUC = 98.50%, IoU = 80.00%
Rosa S. et al. (2024) [63]	**Original image:** pACC = 99.41%, PC = 83.38%, SN = 75.57%, IoU = 68.15%, DSC = 76.58%; **MF:** k = 3: pACC = 99.38%, PC = 81.10%, SN = 74.93%, IoU = 66.64%, DSC = 75.18%; **GF:** k = 3: pACC = 99.37%, PC = 80.61%, SN = 75.04%, IoU = 66.77%, DSC = 75.18%; k = 5: pACC = 99.41%, PC = 83.46%, SN = 73.30%, IoU = 67.11%, DSC = 75.55%; k = 7: pACC = 99.41%, PC = 83.40%, SN = 73.33%, IoU = 66.88%, DSC = 75.35%; **AF:** K = 5, n = 20: pACC = 99.39%, PC = 82.03%, SN = 74.00%, IoU = 66.80%, DSC = 75.31%; K = 5, n = 150: pACC = 99.39%, PC = 81.67%, SN = 72.14%, IoU = 65.45%, DSC = 73.77%; K = 7, n = 150: pACC = 99.32%, PC = 79.68%, SN = 70.82%, IoU = 63.39%, DSC = 71.92%; K = 10, n = 150: pACC = 99.32%, PC = 79.11%, SN = 69.55%, IoU = 62.58%, DSC = 71.01%; K = 13, n = 150: pACC = 99.31%, PC = 78.20%, SN = 68.55%, IoU = 61.65%, DSC = 70.23%; **BF:** σr = 3: pACC = 99.40%, PC = 81.68%, SN = 74.37%, IoU = 66.37%, DSC = 75.07%; σr = 5: pACC = 99.40%, PC = 83.52%, SN = 75.63%, IoU = 68.49%, DSC = 76.96%; σr = 7: pACC = 99.39%, PC = 82.79%, SN = 74.40%, IoU = 67.22%, DSC = 75.76%; σr = 15: pACC = 99.39%, PC = 80.73%, SN = 73.24%, IoU = 65.61%, DSC = 74.01%; **Image with highest IoU:** Original image: pACC = 99.90%, PC = 96.89%, SN = 98.98%, IoU = 95.94%, DSC = 97.93%; MF k = 3: pACC = 99.94%, PC = 98.51%, SN = 97.28%, IoU = 96.46%, DSC = 97.89%; GF k = 5: pACC = 99.95%, PC = 97.64%, SN = 98.75%, IoU = 95.87%, DSC = 98.20%; AF K = 5, n = 20: pACC = 99.95%, PC = 97.87%, SN = 98.73%, IoU = 96.54%, DSC = 97.88%; BF σr = 5, σd = 5: pACC = 99.96%, PC = 98.42%, SN = 98.42%, IoU = 97.21%, DSC = 98.59%
Lefkovits S. et al. (2022) [47]	**BG:** FCN-ResNet50: DSC = 99.65%; Optimized FCN-ResNet50: DSC = 99.82%; FCN-ResNet101: DSC = 99.69%; PSP-ResNet50: DSC = 99.69%; PSP-ResNet101: DSC = 99.61%; DeepLab-ResNet50: DSC = 99.29%; DeepLab-ResNet101: DSC = 99.67%; Ensemble: DSC = 99.58%; **HT:** FCN-ResNet50: DSC = 97.30%; Optimized FCN-ResNet50: DSC = 97.90%; FCN-ResNet101: DSC = 97.40%; PSP-ResNet50: DSC = 97.46%; PSP-ResNet101: DSC = 97.01%; DeepLab-ResNet50: DSC = 95.99%; DeepLab-ResNet101: DSC = 97.26%; Ensemble: DSC = 95.85%; **WT:** FCN-ResNet50: DSC = 90.68%; Optimized FCN-ResNet50: DSC = 91.00%; FCN-ResNet101: DSC = 89.80%; PSP-ResNet50: DSC = 90.09%; PSP-ResNet101: DSC = 89.11%; DeepLab-ResNet50: DSC = 88.89%; DeepLab-ResNet101: DSC = 90.30%; Ensemble: DSC = 87.80%; **ED:** FCN-ResNet50: DSC = 69.63%; Optimized FCN-ResNet50: DSC = 70.46%; FCN-ResNet101: DSC = 71.50% PSP-ResNet50: DSC = 65.89%; PSP-ResNet101: DSC = 75.21%; DeepLab-ResNet50: DSC = 67.40%; DeepLab-ResNet101: DSC = 69.76%; **AT:** FCN-ResNet50: DSC = 69.70%; Optimized FCN-ResNet50: DSC = 71.04%; FCN-ResNet101: DSC = 71.26%; PSP-ResNet50: DSC = 71.21%; PSP-ResNet101: DSC = 69.87%; DeepLab-ResNet50:DSC = 67.80%; DeepLab-ResNet101: DSC = 73.70%; Ensemble: DSC = 76.71%; **NCR/NET:** FCN-ResNet50: DSC = 46.17% Optimized FCN-ResNet50: DSC = 47.40%; FCN-ResNet101: DSC = 44.35%; PSP-ResNet50: DSC = 43.26%; PSP-ResNet101: DSC = 39.49%; DeepLab-ResNet50: DSC = 38.59%; DeepLab-ResNet101: DSC = 47.63%; Ensemble: DSC = 50.08%; **ET:** FCN-ResNet50: DSC = 79.89%; FCN-ResNet101: DSC = 78.73%; PSP-ResNet50: DSC = 78.00%; PSP-ResNet101: DSC = 75.83%; DeepLab-ResNet50: DSC = 79.90%; DeepLab-ResNet101: DSC = 78.79%; Ensemble: DSC = 80.04%; **CT:** FCN-ResNet50: DSC = 84.13%, FCN-ResNet101: DSC = 84.61%; PSP-ResNet50: DSC = 83.55%; PSP-ResNet101: DSC = 83.94%; DeepLab-ResNet50: DSC = 82.77%; DeepLab-ResNet101: DSC = 84.90%; Ensemble: DSC = 85.99%
Koteswara R.C. et al. (2022) [46]	**BraTS 2018:** MCC U-Net + BN: WT HGG: DSC = 88.40%, IoU = 79.21%, SN = 89.72%, SP = 99.64%, pACC = 90.24%; CT HGG: DSC = 81.39%, IoU = 68.62%, SN = 89.72%, SP = 99.64%, pACC = 90.24%; ET HGG: DSC = 76.57%, IoU = 62.04%, SN = 83.12%, SP = 90.14%, pACC = 76.25%; WT LGG: DSC = 67.43%, IoU = 62.87%, SN = 88.69%, SP = 99.01%, pACC = 89.50%; CT LGG: DSC = 64.26%, IoU = 61.54%, SN = 81.53%, SP = 89.13%, pACC = 79.25%; ET LGG: DSC = 64.32%, IoU = 61.03%, SN = 81.87%, SP = 88.23%, pACC = 74.92%; MCC U-Net + GN; WT HGG: DSC = 88.64%, IoU = 79.60%, SN = 92.29%, SP = 99.47%, pACC = 91.64%; CT HGG: DSC = 82.21%, IoU = 69.80%, SN = 88.42%, SP = 93.49%, pACC = 83.72%; ET HGG: DSC = 76.88%, IoU = 62.44%, SN = 82.61%, SP = 90.14%, pACC = 76.25%; WT LGG: DSC = 78.12%, IoU = 77.94%, SN = 90.19%, SP = 99.63%, pACC = 89.92%; CT LGG: DSC = 75.98%, IoU = 68.13%, SN = 82.62%, SP = 90.39%, pACC = 89.92%; ET LGG: DSC = 73.31%, IoU = 61.95%, SN = 82.71%, SP = 89.94%, pACC = 75.00%; MAC U-Net + BN: WT HGG: SC = 92.41%**,** IoU = 84.71%, SN = 92.45%, SP = 99.47%, pACC = 99.02%; CT HGG: DSC = 83.49%, IoU = 71.82%, SN = 89.84%, SP = 93.52%, pACC = 84.73%; ET HGG: DSC = 80.15%, IoU = 65.14%, SN = 82.84%, SP = 91.43%, pACC = 78.01%; WT LGG: DSC = 83.18%, IoU = 83.36%, SN = 90.68%, SP = 98.56%, pACC = 89.80%; CT LGG: DSC = 76.40%, IoU = 70.96%, SN = 84.93%, SP = 91.80%, pACC = 82.69%; ET LGG: DSC = 73.33%, IoU = 62.66%, SN = 80.92%, SP = 90.23%, pACC = 76.83%; MAC U-Net + GN: WT HGG: DSC = 94.47%, IoU = 89.54%, SN = 93.05%, SP = 99.20%, pACC = 92.14%; CT HGG: DSC = 84.12%, IoU = 77.83%, SN = 89.90%, SP = 93.44%, pACC = 87.63%; ET HGG: DSC = 82.72%, IoU = 67.68%, SN = 83.10%, SP = 93.34%, pACC = 77.64%; WT LGG: DSC = 85.71%, IoU = 84.85%, SN = 91.16%, SP = 98.83%, pACC = 90.10%; CT LGG: DSC = 78.75%, IoU = 71.32%, SN = 84.12%, SP = 92.56%, pACC = 84.31%; ET LGG: DSC = 74.16%, IoU = 66.33%, SN = 80.86%, SP = 90.87%, pACC = 75.53%; **BraTS 2019**: MCC U-Net + BN: WT HGG: DSC = 88.45%, IoU = 79.43%, SN = 89.81%, SP = 99.27%, pACC = 85.19%; CT HGG: DSC = 80.69%, IoU = 68.70%, SN = 82.10%, SP = 92.48%, pACC = 80.38%; ET HGG: DSC = 76.72%, IoU = 62.30%, SN = 81.63%, SP = 89.31%, pACC = 76.09%; WT LGG: DSC = 67.43%, IoU = 62.87%, SN = 88.69%, SP = 99.01%, pACC = 89.50%; CT LGG: DSC = 65.52%, IoU = 62.04%, SN = 81.63%, SP = 89.63%, pACC = 77.86%; ET LGG: DSC = 66.02%, IoU = 61.96%, SN = 81.87%, SP = 87.29%, pACC = 74.89%; MCC U-Net + GN: WT HGG: DSC = 88.56%, IoU = 81.12%, SN = 89.89%, SP = 99.33%, pACC = 87.27%; CT HGG: DSC = 80.84%, IoU = 69.04%, SN = 86.20%, SP = 92.64%, pACC = 82.19%; ET HGG: DSC = 81.12%, IoU = 62.43%, SN = 81.76%, SP = 89.41%, pACC = 73.13%; WT LGG: DSC = 76.62%, IoU = 78.38%, SN = 88.10%, SP = 98.88%, pACC = 86.12%; CT LGG: DSC = 76.18%, IoU = 66.72%, SN = 81.76%, SP = 90.19%, pACC = 79.31%; ET LGG: DSC = 70.86%, IoU = 62.44%, SN = 81.81%, SP = 88.28%, pACC = 75.13%; MAC U-Net + BN: WT HGG: DSC = 92.86%, IoU = 85.69%, SN = 94.15%, SP = 99.20%, pACC = 92.07%; CT HGG: DSC = 83.09%, IoU = 72.56%, SN = 89.90%, SP = 93.81%, pACC = 86.13%; ET HGG: DSC = 81.85%, IoU = 66.46%, SN = 83.04%, SP = 91.19%, pACC = 78.10%; WT LGG: DSC = 85.18%, IoU = 83.80%, SN = 91.77%, SP = 99.10%, pACC = 90.48%; CT LGG: DSC = 77.74%, IoU = 71.49%, SN = 84.93%, SP = 92.34%, pACC = 83.09%; ET LGG: DSC = 74.93%, IoU = 91.77%, SN = 99.10%, SP = 90.9%, pACC = 77.30%; MAC U-Net + GN: WT HGG: DSC = 94.75%, IoU = 86.49%, SN = 95.13%, SP = 99.60%, pACC = 92.75%; CT HGG: DSC = 84.23%, IoU = 74.42%, SN = 89.87%, SP = 94.57%, pACC = 88.33%; ET HGG: DSC = 82.84%, IoU = 68.81%, SN = 84.72%, SP = 93.09%, pACC = 78.47%; WT LGG: DSC = 86.56%, IoU = 85.22%, SN = 93.86%, SP = 99.18%, pACC = 91.84%; CT LGG: DSC = 80.85%, IoU = 72.41%, SN = 86.42%, SP = 93.65%, pACC = 85.81%; ET LGG: DSC = 75.83%, IoU = 66.02%, SN = 83.71%, SP = 91.94%, pACC = 77.93%; **BraTS 2020**: MCC U-Net + BN: WT: DSC = 83.47%, IoU = 75.83%, SN = 86.22%, SP = 98.28%, pACC = 82.68%; CT: DSC = 73.91%, IoU = 65.18%, SN = 82.43%, SP = 92.67%, pACC = 79.05%; ET: DSC = 70.72%, IoU = 62.43%, SN = 79.17%, SP = 90.93%, pACC = 74.46%; MCC U-Net + GN: WT: DSC = 83.90%, IoU = 77.41%, SN = 88.96%, SP = 98.80%, pACC = 86.50%; CT: DSC = 76.25%, IoU = 67.62%, SN = 83.77%, SP = 93.28%, pACC = 81.27%; ET: DSC = 74.63%, IoU = 62.58%, SN = 80.58%, SP = 91.59%, pACC = 74.83%; MAC U-Net + BN: WT: DSC = 89.28%, IoU = 82.26%, SN = 92.82%, SP = 99.39%, pACC = 90.91%; CT: DSC = 82.70%, IoU = 72.70%, SN = 88.64%, SP = 94.45%, pACC = 85.27%; ET: DSC = 80.18%, IoU = 66.54%, SN = 83.13%, SP = 92.88%, pACC = 77.19%; MAC U-Net + GN: WT: DSC = 90.45%, IoU = 86.59%, SN = 94.73%, SP = 99.30%, pACC = 91.49%; CT: DSC = 84.40%, IoU = 74.38%, SN = 89.40%, SP = 92.70%, pACC = 86.29%; ET: DSC = 82.16%, IoU = 68.45%, SN = 84.68%, SP = 92.70%, pACC = 78.58%
Sachdeva J. et al. (2024) [45]	**BraTS 2020:** ET: DSC = 81.00%, SN = 91.00%, SP = 98.00%, HD95 = 4.22; WT: DSC = 91.00%, SN = 95.00%, SP = 99.00%, HD95 = 4.44; CT: DSC = 83.00%, SN = 97.00%, SP = 99.00%, HD95 = 6.72; **BraTS 2021:** ET: DSC = 86.00%, SN = 94.00%, SP = 100.00%, HD95 = 3.64; WT: DSC = 92.00%, SN = 97.00%, SP = 100.00%, HD95 = 4.11; CT: DSC = 84.00%, SN = 97.00%, SP = 100.00%, HD95 = 5.47; **Real-time clinical data from PGIMER:** ET: DSC = 79.00%, SN = 93.00%, SP = 99.00%, HD95 = 15.12; WT: DSC = 76.00%, SN = 90.00%, SP = 99.00%, HD95 = 16.08; CT: DSC = 68.00%, SN = 88.00%, SP = 99.00%, HD95 = 18.14
Yang L. et al. (2025) [44]	**BraTS 2018:** ET: DSC = 81.50%, HD95 = 3.49; WT: DSC = 90.10%, HD95 = 6.24; CT: DSC = 81.50%, HD95 = 6.15; **BraTS 2020:** ET: DSC = 83.50%, HD95 = 2.42; WT: DSC = 91.50%, HD95 = 3.76; CT: DSC = 82.30%, HD95 = 6.44; **TCGA-LGG:** k-Cohen = 0.68, DSC = 70.20%, IoU = 58.10%, SN = 65.80%, SP = 76.20%, PC = 76.20%, pACC = 98.10%, bACC = 80.80%, AUC = 81.20%
Zaitoon R. et al. (2023) [43]	**HGG:** NCR: pACC = 97.97, DSC = 98.39%; ED: pACC = 98.24, DSC = 99.10; ET: pACC = 98.36%, DSC = 98.69%; NET: pACC = 98.73%, DSC = 98.20%; **LGG:** NCR: pACC = 98.15%, DSC = 98.32%; ED: pACC = 98.68%, DSC = 98.94%; ET: pACC = 99.11%, DSC = 98.94%; NET: pACC = 99.54%, DSC = 99.41%
Rahat I.S. et al. (2023) [59]	3D U-Net: DSC = 35.04%; DeepLabv1: DSC = 84.13%; DeepLabv2: DSC = 80.68%; DeepLabv3: DSC = 81.77%; DeepLabv3+: DSC = 65.17%; DenseNet121 U-Net: DSC = 85.23%, IoU = 74.23%; ResNet50: TI = 90.35%; Attention U-Net: DSC = 27.57%, IoU = 16.21%; EfficientNet: DSC = 92.37%, IoU = 89.55%

Legend: nMN: non-malignant neoplasm, HT: Healthy Tissue, AT: Active tumor, BG: Background, WT: whole Tumor, NCR: Necrotic Tumor Region, NET: Non-enhancing tumor regions, ED: Edematous tissue, CT: Core Tumor, ET: Enhancing tumor regions, DSC: Dice Score Coefficient, LGG: Low grade glioma, HGG: High grade glioma, BTF: Brain Tumor Figshare, K-BT: Kaggle Brain Tumor dataset, K-BTC: Kaggle Brain Tumor classification dataset, TCGA-LGG: The Cancer Genome Atlas Low-Grade Glioma, TC: tight cropping, MF: median filter, GF: gaussian filter, AF: anisotropic filter, BF: bilateral filter, ep: epochs, dp: drop-out rate, HD95: 95th percentile Hausdorff Distance, pACC: pixel accuracy, MCCoef: Matthews Correlation Coefficient, AUC: Area Under the Curve, V-DSC: Volumetric-DSC, S-DSC: Surface-DSC, IoU: Intersection over Union, AFMS-SAEB: Adaptive Feature Medical Segmentation-Single Adaptive Encoder Block, AFMS-DAEB: Adaptive Feature Medical Segmentation-Dual Adaptive Encoder Block, BTS: Brain Tumor Segmentation, SEL: stacking ensemble learning, VGG: visual geometry group, k: kernel size, K: gradient sensibility, n: number of iterations, σr: controls fall-off of the weights in spatial and intensity domains, FCN: Fully Convolutional Network MAC: Multimodal attention-gated cascaded, MCC: Multi-Channel cascaded, BN: Batch Normalization, GN: Group Normalization, PGIMER: Post Graduate Institute of Medical Education & Research, Chandigarh, SP: specificity, SN: sensitivity, PC: Precision, bACC: Balanced Accuracy, TI: Tversky index.

#### 3.2.2. Brain Tumor Classification

Table 3 summarizes the studies that performed segmentation followed by classification, thereby providing additional information on the classification task. In particular, the feature extraction method, the features used for the classification, the feature selection algorithm applied (if any), the training/validation/testing splits, the presence of external validation or fine-tuning datasets, the model hyperparameters, the target of the classification task (i.e., the predicted classes), the performance metrics used for classification, the application of XAI methods, and the major findings obtained. Performance metrics are better explained in the Supplementary Materials (see Table S2).

Among the 31 studies identified as eligible for the review, eight focused on both brain tumor segmentation and classification [18,37,38,43,57,60,61,65], while the remaining studies addressed only segmentation. Among these studies, two included both patients and controls [37,38]. Regarding tumor type, three studies focused on glioma [18,39,53], in particular HGG and LGG; four studies investigated glioma, meningioma, and pituitary tumors [38,60,61,65]; and only one study considered diverse tumor cases, including brainstem glioma [37]. To perform segmentation and classification tasks, two studies used only the BTF dataset [60,61], three used BraTS from 2017 to 2020 [18,43,57], one used both BTF and BraTS 2018 [65], another one used the Kaggle Brain Tumor MRI Dataset [38], and the last one used a custom-made, not publicly available dataset [37].

As previously demonstrated, studies used a single dataset or a combination of datasets, with a median sample size of 301, a minimum of 94 subjects, and a maximum of 3264 subjects. All selected papers used MRI, though the specific modalities varied across studies. Three studies employed T1ce-weighted MRI [60,61,65], three other studies used mpMRI, i.e., FLAIR, T1-weighted, T1ce-weighted and T2-weighted [18,43,57], and one study also added DWI sequence [37]. Only one study did not specify which sequences were used [38].

In seven of eight papers, features for training the classification model were extracted using a CNN [37,38,43,57,60,61,65]. Maqsood S. et al. (2022) [65] used MobileNetV2 [79], a CNN model with inverted residuals and linear bottlenecks, to extract low-, middle-, and high-level features for classification; the extracted features were subsequently refined through feature selection using an entropy-controlled method. The other six studies employed a single CNN-based DL model to perform both segmentation and classification, leveraging the same feature representations extracted by the CNN for training and prediction in both tasks [37,38,43,57,60,61]. Among these, one study combined CNN features with clinical information solely for classification [37]. Furthermore, Aumente-Maestro et al. (2025) [18], after the segmentation extracted radiomics features, that were clustered in MRI features, i.e., mean, standard deviation, minimum and maximum pixel intensity, skewness, kurtosis, entropy, and blurriness, and features mainly related to the segmented mask called segmented, i.e., the number of pixels assigned to each segmented area, the tumor size and extension along each of the X, Y, and Z axes.

The validation strategies reflected different levels of rigor in model evaluation. Specifically, five studies used a hold-out validation approach, with different percentages for the train, validation, and test sets [37,38,43,57,60]. More rigorous validation was demonstrated in two studies [18,61], which used the 5-fold cross-validation. Just one study combined the two approaches [65]. Most classification studies reported high performance on their own dataset, but no evidence of external validation or fine-tuning.

Across the reviewed classification studies, four focused on predicting primary tumor types, such as glioma, meningioma, and pituitary tumors [38,60,61,65], whereas three focused on predicting glioma grading (HGG vs. LGG) [18,43,57]. One study [37] expanded the classification scope beyond the traditional three classes to include Ependymoma, medulloblastoma, brainstem glioma and pilocytic astrocytoma.

To perform the classification task, 32 studies used an ML model. In particular, Maqsood et al. (2022) [65] used a multiclass SVM to predict the tumor type, reaching on BraTS 2018 dataset an accuracy of 97.47%, 97.22% sensitivity, and 97.94% specificity on the BraTS 2018 dataset, and an accuracy of 98.92%, 98.82% sensitivity, and 99.02% specificity on the BTF dataset. Aumente-Maestro C. et al. (2025) [18] used an XGBoost model to predict the glioma stage (i.e., HGG vs. LGG). In particular, the authors trained three different models: (i) using only MRI features; (ii) using only segmented features; and (iii) the two categories combined. The best model was the third one, which achieved the highest accuracy equal to 93.20%, 97.30% sensitivity, and 77.60% specificity; followed by the first model, which achieved an accuracy of 91.30%, 96.60% sensitivity, and 70.90% specificity, and lastly the second one showing an accuracy of 81.30%, 91.10% sensitivity, and 43.40% specificity. As XGBoost inherently provides a score for each input feature reflecting its contribution to the prediction, this study was the only one to conduct an explainability analysis showing that the number of voxels in ET, the number of voxels in CT, and kurtosis were the most important features.

The remaining studies used DL models to perform both segmentation and classification. Rai et al. (2024) [60], using the two-headed UNetEfficientNets, showed that B0 achieved a global sensitivity of 97.00% and an accuracy of 94.80%, with tumor-specific sensitivities of 92% for meningioma, 99% for glioma, and 100% for pituitary tumors. B1 improved performance further, reaching a global 97.67% sensitivity and 99.00% accuracy, with meningioma at 94% sensitivity, glioma at 99% sensitivity, and pituitary tumors at 100%. B2 showed slightly lower global sensitivity (i.e., 81.67%) but maintained 93.00% accuracy, with tumor-specific metrics of 67% sensitivity for meningioma, 98% for glioma, and 80% for pituitary tumors. B3 achieved a global sensitivity of 98% and 96% accuracy, with meningioma at 95% sensitivity, glioma at 99% sensitivity, and pituitary tumors at 100% sensitivity. B4, the best-performing variant, achieved a global sensitivity of 99.00% and 99.40% accuracy, with tumor-specific sensitivity of 98% for meningioma, 99% for glioma, and 100% for pituitary tumors. Finally, B5 showed a global sensitivity of 97.67% and 99.30% accuracy, with meningioma at 94% sensitivity, glioma at 99% sensitivity, and pituitary tumors at 100% sensitivity.

Sobhaninia et al. (2023) [61] used a Multiscale Cascaded Multitask Network, as previously mentioned, created to solve both segmentation and classification tasks. In performing tumor type classification using the Multiscale Cascaded Multitask Network, they achieved a global accuracy of 95.09%. When the aggregation module was included in the model, the global accuracy increased to 97.99%. Focusing on the single tumor type, the highest precision was achieved for glioma (precision = 98.32%), followed by meningioma and pituitary tumor (precision = 97.60% and 97.10%, respectively).

Krishnasamy et al. (2023) [38] introduced two hybrid models for brain tumor segmentation and tumor type classification, as stated before. ResNet-50, a deep convolutional neural network, is based on a residual learning framework that mitigates the vanishing gradient problem, enabling the training of very deep networks [80]. MobileNet, in contrast, is a lightweight model optimized for mobile and embedded vision applications, with efficiency that makes it particularly suitable for real-time scenarios with limited computational resources [81]. In terms of performance, Model 1 achieved 94.00% of sensitivity, an accuracy of 98.93%, and a specificity at 98.70%. Model 2 showed similar performance, with a sensitivity of 92.00%, an accuracy of 98.63%, and a specificity of 98.50%.

Lastly, Mahajan et al. (2023) [37] evaluated the classification of several pediatric brain tumor types, although the specific AI model used was not reported. For brainstem gliomas, the model achieved an accuracy of 94.29%, sensitivity of 88.24%, and specificity of 96.23%. Ependymomas were classified with 90.00% accuracy, 64.29% sensitivity, and 96.43% specificity. For medulloblastomas, performance reached 98.57% accuracy, 61.90% sensitivity, and 100% specificity. Finally, pilocytic astrocytomas showed 84.29% accuracy, 100% sensitivity, and 78.85% specificity.

Focusing on glioma staging classification, Abd-Ellah et al. (2024) [57] used a two-pathway residual-based deep convolutional neural network architecture (TRDCNN) comprising four main paths: global, local, merged, and output. The global and local paths independently extract features using convolutional, ReLU, and max-pooling layers, followed by six residual block stages. Operating in parallel, the two paths are later combined into a merged path comprising batch normalization, ReLU, fully connected, and dropout layers. Finally, the output path includes a softmax classification layer that classifies HGG vs. LGG images, achieving an accuracy of 98.88%, specificity of 99.00%, and sensitivity of 98.66%.

Zaitoon and Syed (2023) [43] employed the Deep Brain Tumor Convolutional Neural Network (DBT-CNN) for glioma staging classification using the segmented output from RRU-Net2+. DBT-CNN consists of convolutional, pooling, activation, and fully connected layers, with three convolutional layers followed by layer normalization, ReLU activation, three max-pooling layers, a dropout layer, and a final softmax layer. Classification was performed both on the entire mask and on precisely extracted tumor areas. The model achieved high performance for both tumor types. For HGG, accuracy was 98.51 ± 0.18%, 97.23 ± 0.28% sensitivity, and 98.62 ± 0.46% specificity. For LGG, the accuracy was 99.28 ± 0.18%, 97.83 ± 1.22% sensitivity, and 98.88 ± 0.16% specificity.

To summarize, the average accuracy for tumor type classification was 96.95%, with a range of 91.79% to 99.40%. Regarding glioma staging, the average accuracy was 95.97%, with a range of 91.30% to 98.90%.

**Figure 3:**
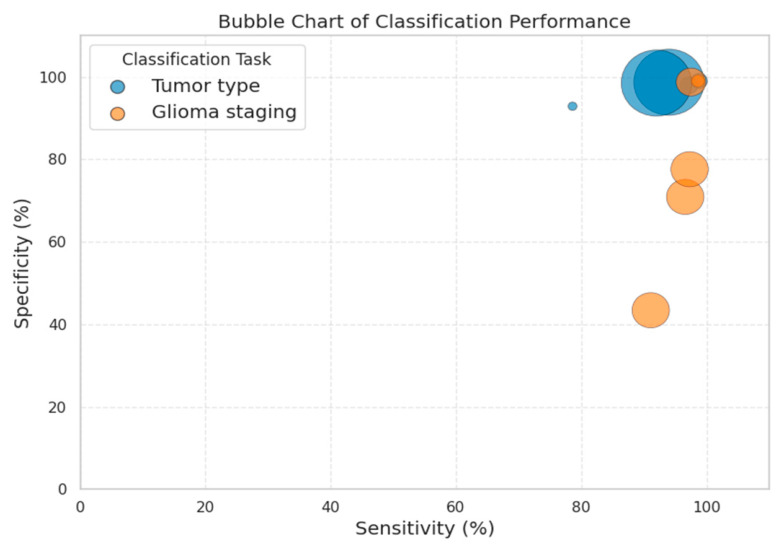
The bubble chart illustrates the distribution of models by sensitivity (*x*-axis) and specificity (*y*-axis), plotted when reported. Each bubble represents a model, with its size proportional to the test sample size and color indicating the classification task. Models located in the upper right corner exhibit both high sensitivity and specificity and thus correspond to the best-performing approaches. Conversely, models in the upper-left show high specificity but low sensitivity, while those in the bottom-right display high sensitivity but lower specificity. This visualization enables immediate comparison of performance across different classification tasks when these metrics are available.

Figure 3 presents a bubble chart illustrating the distribution of model performance, where the *x*-axis represents sensitivity, the *y*-axis represents specificity, and the bubble size reflects test size. This Figure shows that most of the models reported in the selected papers exhibited either high sensitivity or high specificity, and only one paper reported higher sensitivity and lower specificity, with no influence of the sample size used in the testing phase.

**Figure 4:**
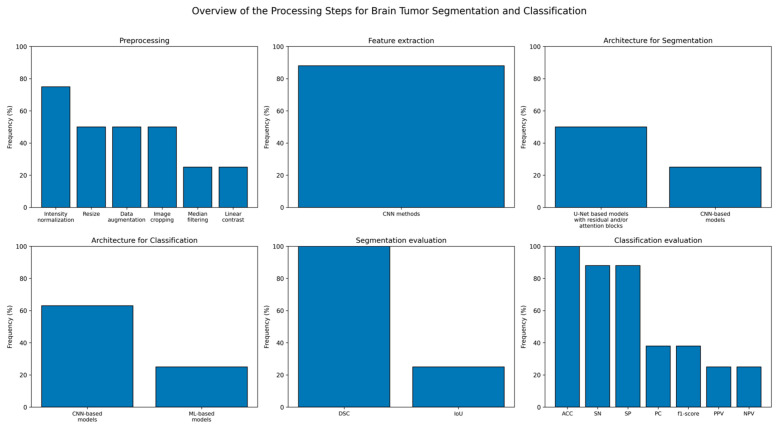
Summary of the methodological pipeline used by studies included in this review that tackled both brain tumor segmentation and classification. The relative frequency of approaches adopted for each step is reported. Legend: CNN: convolutional neural network, ML: machine learning, ACC: accuracy, SN: sensitivity, SP: specificity, PC: precision, PPV: positive predictive value, NPV: negative predictive value.

To facilitate interpretation of the results, Figure 4 summarizes the complete methodological pipeline adopted by the eight selected studies for segmentation and classification tasks, from preprocessing through model architecture to performance evaluation. Only methods reported in at least 25% of the included studies were considered.

In the preprocessing phase, 75% of the studies performed intensity normalization, while 50% applied image cropping, resizing, and data augmentation. Linear contrast augmentation and median filtering were adopted by 25% of the studies each. Regarding feature extraction, 88% of the selected studies employed CNN-based methods. For segmentation, 50% of the studies used U-Net–based architectures incorporating residual blocks and/or attention mechanisms and 25% traditional CNN models. For classification, 63% of the studies adopted CNN models, whereas 25% used ML approaches, such as Random Forest and XGBoost. Regarding model evaluation, all studies assessed segmentation performance using DSC, and 25% also reported IoU. For classification, all studies reported accuracy; 88% reported sensitivity and specificity, 38% reported precision and F1-score, and 25% reported positive predictive value (PPV) and negative predictive value (NPV).

### 3.3. Risk of Bias Within Studies

#### 3.3.1. Brain Tumor Segmentation

**Figure 5:**
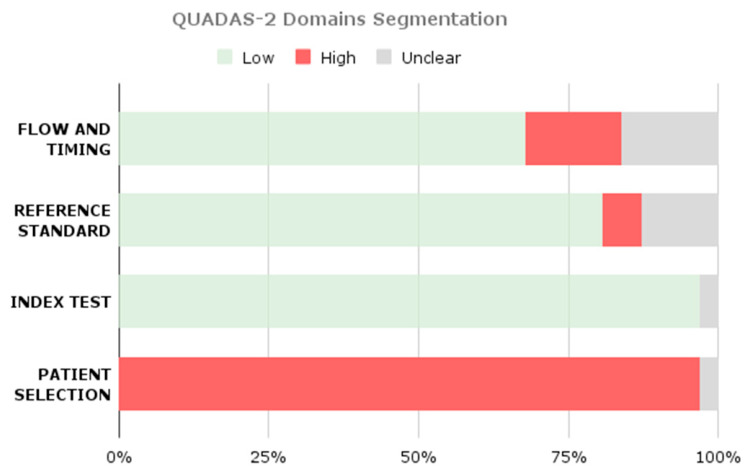
Proportion of included studies categorized by risk of bias assessment (low, high, or unclear).

The risk of bias in the included studies, along with the authors’ comments on the seven QUADAS-2 domains, was evaluated. Figure 5 presents the assessment results across these domains for all studies included in the review. Almost all studies (30 out of 31) showed low concerns regarding applicability, as the patient characteristics, study setting, conduct and interpretation of the index test, and the target condition defined by the reference standard were all aligned with the review question. Only one study was rated as having unclear concern because it did not report the validation method used to train the DL model, making it impossible to determine whether there was any overlap between patients used for training and testing [37]. Looking at the other domains, most studies used publicly available datasets that did not enroll patients consecutively or randomly. Only five studies used custom-made datasets, as previously mentioned, in which patients were retrospectively recruited, or, in one case, provided no information on how they enrolled patients [37,40,41,42,45]. Regarding the reference standard domain, twenty-five studies were rated as having low risk of bias, as the reference segmentations were either manually generated by three or more experienced clinicians, produced by trained medical students and subsequently verified by experts, or initially generated by an algorithm and then reviewed by clinicians [17,18,40,43,44,45,46,47,48,49,50,51,53,54,55,56,57,58,59,60,61,62,63,64,65]. Four studies were rated as having unclear risk due to a lack of information on clinicians’ experience levels or because segmentation was performed by fewer than three operators [38,39,41,52]. Two studies were rated as high risk because the segmentations were performed by only one experienced neurologist [37,42]. Lastly, in the flow and timing domain, twenty-one studies were rated as having low risk of bias [18,37,38,40,41,42,46,48,49,50,51,52,54,55,56,60,61,62,63,64,65]. Five studies were rated as high risk due to the exclusion of some patients from the analysis [42,53,56,57,58]. In contrast, another five were rated as unclear risk because the number of patients included in the analysis was not reported [17,39,44,45,47].

The risk of bias tools highlighted the following limitations:

- No study used consecutive or random recruitment. Only three studies used a custom-made dataset for the analysis [37,40,42], but only two studies specified that patients were retrospectively recruited [40,42];
- Few studies [17,39,42,44,45,47,53,56,57] did not report information regarding possible inappropriate exclusion of patients, which could introduce selection bias into the analyzed sample;
- The exclusion of patients from the final analysis in a subset of studies [37,42,53,56,57] or the lack of information about the final sample [17,39,44,45,47] used for the analysis could lead to attrition bias, affecting the generalizability of results;
- In at least one study [65], the absence of details on the validation approach used for training the DL model makes it impossible to assess the risk of overfitting and whether the training and testing datasets were truly independent.

#### 3.3.2. Brain Tumor Classification

**Figure 6:**
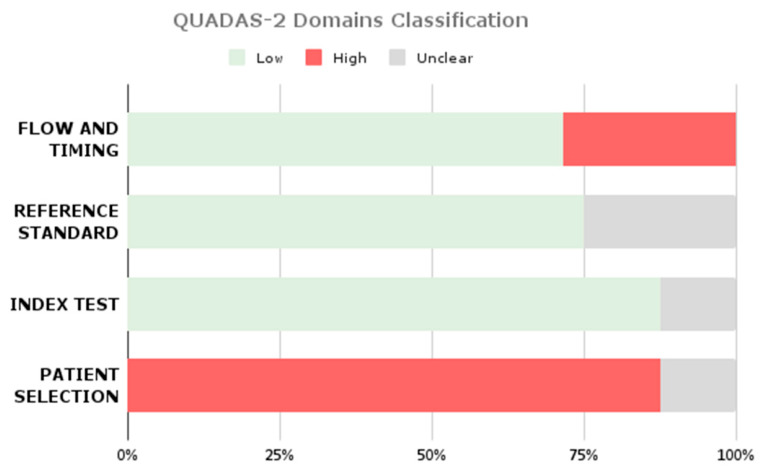
Proportion of included studies that performed both segmentation and classification, categorized by risk of bias assessment (low, high, or unclear).

The risk of bias in the included classification studies was evaluated using the QUADAS-2 framework. Figure 6 summarizes the distribution of judgments across the four domains. Overall, five studies showed low concerns regarding applicability, while three showed great concerns. In the patient selection domain, all studies relied exclusively on publicly available datasets (BTF, BraTS, Kaggle, TCGA) that were not based on consecutive or random patient recruitment, except for one study [37], resulting in a generally high risk of bias. For the index test domain, most studies were rated as low risk. Classification thresholds were not explicitly reported, and threshold selection procedures were not described [17,18,37,38,39,43,57,60,61,62,63,64,65]. Three studies [18,61,65] employed robust cross-validation strategies and explicit training, validation, and testing splits and were therefore judged to be at low risk of bias. In the reference standard domain, studies using BraTS and BTF datasets were considered to have a low risk of bias [18,38,39,60,61,62,63,64,65]. In contrast, two studies [33,34] were judged to have unclear risk. Finally, the flow and timing domain showed mixed results: most studies applied consistent reference standards and clearly reported dataset splits, leading to low-risk judgments. In contrast, two studies [37,57] were rated as having a high risk of bias.

The risk-of-bias analysis highlighted the following limitations:

- Except for one study [37] that is unclear, no other work employed consecutive or random recruitment, but relied on a curated public dataset that led to a high risk of bias;
- Thresholds for classification and validation approaches were not prespecified in any study, thus introducing potential bias in index test performance [18,37,38,43,57,60,61,65];
- Patient-selection bias was generally high, while it was judged as unclear in one case [41], due to uncertain sampling methods and inappropriate exclusion criteria;
- Bias related to flow and timing was generally low, although two studies [41,60] showed high risk due to incomplete inclusion of all patients or unclear reporting of analysis steps.

## 4. Discussion

This review aimed to examine the existing literature on DL-based approaches for the automatic segmentation of gliomas, and, when performed, to analyze the AI methods used for automatic tumor type classification or glioma staging. Overall, the reviewed studies show that CNNs, particularly U-Net-based architectures, dominate glioma segmentation tasks. For the classification task, CNN-based and hybrid models demonstrate promising performance. Evidence on XAI analyses remains scarce in both segmentation and classification studies.

### 4.1. Tumor Heterogeneity and Challenges in Segmentation

Meningiomas are generally easier to segment due to their well-circumscribed nature, while pituitary tumors present distinct challenges related to their small size, anatomical location, and low tissue contrast. Glioma cases are the most prevalent and the most challenging to segment due to their heterogeneous appearance and diffuse, infiltrative growth patterns. These factors make manual delineation of gliomas not only time-consuming but also subjective and inconsistent [15,67]. Given the critical role of glioma segmentation in treatment planning, surgical navigation, and longitudinal disease monitoring, segmentation accuracy directly influences patient outcomes.

Consistently, this review demonstrates that all studies included glioma cases, and a smaller subset also included meningioma and pituitary tumor cases, underscoring the urgent need for fully automated tools in glioma segmentation. An objective and time-efficient method is needed to reduce interobserver variability and improve clinical workflow efficiency, as highlighted by recent studies [82].

### 4.2. Brain Tumor Regions and Multiparametric MRI

When addressing glioma segmentation, the choice of imaging modality is not merely technical but fundamentally shapes model performance, biological interpretability, and clinical applicability. The mpMRI is widely regarded as the most appropriate imaging strategy in clinical neuro-oncology because it captures complementary structural and physiological information across tumor subregions [83]. This multidimensional characterization is particularly relevant to gliomas, whose infiltrative growth, heterogeneous cellularity, necrosis, and peritumoral edema manifest differently across imaging sequences. Across the studies included in this review, most combined T1-weighted, T2-weighted, T1ce-weighted, and FLAIR sequences to leverage complementary information from different tumor regions and surrounding tissues. Each sequence, in fact, contributes distinct biological information: T1ce enhances visualization of blood–brain barrier disruption and active tumor components; FLAIR improves delineation of peritumoral edema and infiltrative regions; T2-weighted imaging captures fluid-sensitive changes; and T1-weighted imaging provides anatomical reference and structural context. The integration of these modalities allows DL models to exploit cross-sequence intensity relationships and spatial dependencies, which are essential for accurate subregion segmentation.

However, reliance on full mpMRI protocols introduces both clinical and methodological constraints. In routine practice, complete imaging sequences may be unavailable due to protocol variability, scanner differences, time limitations, or retrospective data collection [84,85]. From a computational standpoint, multimodal inputs increase model complexity and amplify susceptibility to inter-scanner variability, intensity non-standardization, and domain-shift effects, potentially limiting robustness beyond harmonized benchmark datasets [86,87].

A small subset of studies restricted segmentation to the WT region, often reporting higher and more stable DSC. In contrast, most studies attempted fine-grained segmentation of multiple glioma subregions, including ET, CT, NCR, NET, and ED. Notably, segmentation performance was consistently lower and more variable for ET and CT compared to WT, reflecting the intrinsic difficulty of delineating smaller, heterogeneous, and less well-defined subregions. This variability highlights an important methodological issue: high overall WT performance may mask suboptimal delineation of clinically critical tumor compartments.

Moreover, inconsistent definitions of tumor subregions across datasets and studies may further limit comparability. While benchmark datasets such as BraTS provide standardized annotations, real-world clinical delineations may differ in granularity and interpretation. Consequently, segmentation models optimized on benchmark definitions may not fully align with clinical decision-making requirements.

Taken together, while mpMRI remains the gold standard for glioma segmentation and underpins the strongest-performing DL architectures, its use does not eliminate methodological challenges. Future research should systematically investigate modality ablation strategies, cross-sequence robustness, and performance under incomplete imaging protocols to better approximate real-world deployment conditions.

### 4.3. Datasets for Brain Tumor Segmentation: Public and Private Sources

Researchers in this field often rely on publicly available datasets collected in multicenter settings, which increase sample size and data diversity. However, these datasets are typically acquired under standardized and harmonized protocols, which reduce technical variability but may not fully capture the heterogeneity of real-world clinical data. This introduces a well-recognized issue of domain shift, whereby models trained on curated benchmark datasets may fail to generalize to data acquired in different clinical settings. Several studies have highlighted that public medical imaging datasets may contain acquisition-specific patterns, preprocessing artifacts, class imbalance, or dataset-specific shortcuts that models can inadvertently exploit, leading to overly optimistic performance estimates and limited generalizability to real-world clinical data, limiting their robustness [88,89].

Conversely, studies based on private, single-center datasets often involve smaller sample sizes and highly specific acquisition settings. Although these data may better reflect local clinical practice, their limited heterogeneity and lack of external validation can lead to an overestimation of model performance [86,90].

Such limitations must be taken into account when developing AI models and evaluating the results of AI-based systems. Reliance on either highly curated public datasets or small, single-center cohorts introduces distinct but equally relevant sources of bias. Importantly, the high performance frequently reported in the literature, such as DSC exceeding 0.90, may reflect optimization within controlled benchmark environments rather than true robustness in clinical practice. From a translational perspective, model reliability cannot be inferred solely from performance on harmonized datasets. Instead, methodological rigor requires systematic cross-domain validation strategies, ideally combining training on diverse public datasets with testing on independent, real-world clinical cohorts [87]. Our review reveals that the majority of included studies relied exclusively on publicly available datasets such as BraTS, BTF, and TCGA-LGG, with only a minority incorporating external validation using independent institutional data. This pattern reflects a broader benchmark-driven optimization trend in the field, in which model development is frequently tailored to standardized challenges rather than to heterogeneous clinical environments. Consequently, the generalizability of many reported DL-based segmentation systems remains uncertain, and performance metrics should be interpreted cautiously when considering clinical deployment.

### 4.4. Training Automated Tools: Current Challenges and Limitations

In a survey by Gordillo et al. (2013) [22], several automatic methods for brain tumor segmentation, including artificial neural networks (ANNs), were identified. While effective, these approaches face challenges related to network complexity, long training times, and the need for large amounts of training data. More recently, a review by Magadza and Viriri (2021) [91] highlighted that the scarcity of large-scale, high-quality medical datasets remains the major limitation to reducing bias and variance in training. In their review, total sample sizes ranged from 50 to 750, with training sets comprising 35 to 484 subjects and testing sets comprising 15 to 266 subjects. Consistently, our analysis shows that patient cohort sizes vary widely across studies, from 65 to over 3000 subjects. While larger datasets can improve model robustness, this variability underscores a critical issue: many segmentation studies remain underpowered, and performance reported on small cohorts may overestimate real-world generalizability, emphasizing the need for standardized, adequately sized datasets for reliable model evaluation.

To address the limited availability of training data, researchers have commonly employed data augmentation or Generative Adversial Networks (GAN) to generate synthetic variations and expand the training set, enhancing model robustness [92]. Our findings confirm that several studies incorporated data augmentation in their preprocessing pipelines, but their limitations must be carefully considered. In particular, synthetic data may not fully capture the underlying biological variability and heterogeneity of gliomas, potentially limiting their clinical relevance. Generated samples can introduce subtle artifacts or amplify dataset-specific biases, leading models to learn patterns that do not correspond to true biological features. As a result, performance improvements obtained using synthetic data may be overestimated, especially when not validated on independent real-world datasets. Furthermore, assessing the biological plausibility of generated data remains challenging, raising concerns about their reliability in clinical applications. Therefore, while these approaches represent valuable tools for mitigating limited data availability, their use should be complemented with rigorous validation and, whenever possible, supported by high-quality, multi-center clinical data.

In addition, the training can be improved through preprocessing steps such as bias field correction, skull stripping, intensity normalization, resizing, and image cropping [81,88,89,93]. Despite these benefits, preprocessing introduces additional computational steps and potential sources of error.

### 4.5. DL Methods for Segmentation

CNNs have become the dominant paradigm for medical image segmentation due to their ability to learn hierarchical spatial features from imaging data. In brain tumor analysis, they are particularly effective in capturing complex intensity patterns and structural variability across MRI modalities. Among these, U-Net and its derivatives constitute the foundational architecture for glioma segmentation, integrating global contextual information with fine structural details necessary to delineate tumor subregions with irregular boundaries and heterogeneous intensities [94]. Performance in challenging regions, including NCR, NET, and ED, is further enhanced by U-Net variants that incorporate residual connections and attention mechanisms, thereby improving feature learning and boundary delineation. Attention-based architectures, such as Attention U-Net and its cascaded variants, also suppress irrelevant background information and emphasize clinically meaningful tumor regions [95].

Consistent with this theoretical framework, our results confirmed the predominance of CNN-based approaches, which demonstrated stable and high performance across studies. U-Net-based models with residual blocks (i.e., 3D RR-UNet2+) achieved higher DSC in LGG cases across NCR, ED, ET, and NET. In contrast, models lacking multi-scale or attention-based strategies, such as 2DVNet or ResNet50, reported substantially lower performance, highlighting the strong association between residual and attention mechanisms and segmentation accuracy. Overall, these findings underscore that careful architectural customization, particularly through the integration of residual and attention modules, remains critical for achieving clinically reliable glioma segmentation.

### 4.6. DL Methods for Classification

Classification is the final stage in the tumor diagnosis pipeline, focusing on differentiation and staging. Accurate and early classification is essential to translate imaging findings into meaningful clinical decisions. The reviewed studies reported overall high performance in tumor type identification, relying on CNNs to jointly perform segmentation and classification. Despite these promising results, the limited use of external testing and the prevalence of hold-out strategies raise concerns about model generalizability.

Regarding glioma staging, only a few studies have focused on distinguishing HGG from LGG and have achieved high performance. To perform this task, CNNs or hybrid DL architectures were employed, whereas one study uniquely combined radiomic and segmentation-derived features within an XGBoost framework, reaching comparable performance metrics.

### 4.7. Trade-Off Between Model Complexity and Clinical Usability in DL Models

This review highlights the rapid evolution of DL, particularly the development of increasingly complex architectures for glioma segmentation, often achieving high performance when trained on large-scale datasets. This trend reflects a shift from conventional CNN-based approaches toward more advanced hybrid and transformer-based models.

However, the growing architectural complexity introduces important limitations that must be carefully considered. The integration of attention mechanisms and residual connections increases the number of trainable parameters, with direct implications for both training and deployment [96]. These models require substantial computational resources, including high memory consumption and increased demand for computational capacity, and often involve longer inference times, factors that may limit their applicability in time-sensitive clinical workflows. In addition, model usability and interpretability remain critical challenges. For clinical adoption, models must not only be accurate but also transparent, reliable, and easy to integrate into routine practice. Increasing complexity often reduces interpretability, potentially limiting clinician trust and effective use in decision-making processes.

Taken together, these findings underscore a key trade-off between model complexity and clinical applicability. While more advanced architectures may provide marginal performance improvements, this review shows that simpler models can also offer more stable and efficient behavior, especially in automatic glioma segmentation. This could favor the integration into real-world clinical settings. In this context, current research is shifting toward the design of lightweight models that maintain competitive performance while enhancing efficiency, interpretability, and real-world deployability [97].

### 4.8. Explainability Analysis

XAI is crucial for the clinical adoption of DL models in both segmentation and classification. With the growing adoption of AI in human-centered contexts, and the increasing societal impact of algorithmic decisions, there has been a marked shift in focus from performance metrics toward explainability [98]. Despite their high performance, many models remain “black boxes,” limiting clinicians’ trust and hindering real-world application [22,99]. This lack of transparency makes it difficult to assess model reliability, increases the risk of overconfidence, and reduces clinical applicability.

XAI aims to make machine decisions understandable and assessable in terms of trustworthiness. It represents a paradigm for bridging machine intelligence and human reasoning, with the goal of improving the acceptance of AI systems in human-centered domains, particularly in healthcare and medicine. In this context, XAI can be viewed as “AI for people.” The need for XAI arises from concerns about deploying highly complex systems without transparent decision-making processes that can be interpreted by domain experts (e.g., clinicians, legal professionals, or financial analysts). Beyond enabling the interpretation of individual decisions, XAI also promotes the development of more human-centered models and contributes to a deeper understanding of cognitive and information-processing mechanisms [100]. Systematic integration of XAI with rigorous validation frameworks could enhance trust and reproducibility, ultimately supporting the implementation of high-performance models in clinical workflows [101].

Several XAI methods have been developed that should be integrated into future studies. These methods can be broadly categorized as local or global, as well as post hoc and model-agnostic. In the context of medical imaging, explainability can be achieved using techniques such as Grad-CAM [102], which generates visual heatmaps highlighting the image regions that most influence the model’s decision. In classification tasks, SHapley Additive exPlanations (SHAP) [103] quantify the contribution of each feature to the prediction. SHAP is particularly valuable as it provides both global and local interpretability by estimating the impact of individual features across the model as a whole and for specific predictions. In contrast, Local Interpretable Model-agnostic Explanations (LIME) [104] focuses on local interpretability by approximating the model behavior in the vicinity of a single prediction.

Our review highlights a clear imbalance between performance optimization and interpretability assessment. Among the segmentation studies, only one employed Grad-CAM to visualize model attention patterns. Similarly, in the classification studies, decision-driving features were rarely reported or formally analyzed; only one study provided an explicit evaluation of feature importance. This limited adoption of XAI methods suggests that explainability is often treated as optional rather than integral to model development.

For DL-based glioma segmentation and classification systems to move beyond benchmark success toward clinical deployment, explainability should be systematically embedded within model design and evaluation pipelines, alongside external validation and robustness testing. Without such integration, even high-performing models risk remaining experimental tools rather than clinically trustworthy systems.

## 5. Conclusions

CNN-based architectures, particularly U-Net variants with residual and attention mechanisms, remain the dominant approach for glioma segmentation, while CNN-based and hybrid models are widely used for tumor classification and grading. Despite high reported performance, their clinical translation remains uncertain.

Most studies rely on harmonized public benchmark datasets and lack independent external validation. Combined with heterogeneous preprocessing, annotation, and evaluation protocols, this limits comparability and may lead to optimistic performance estimates. Additionally, explainability methods remain underutilized.

Future research should prioritize cross-institutional validation using both public multi-center datasets and independent clinical cohorts, alongside standardized evaluation and reporting practices. Integrating imaging with clinical and molecular data and embedding explainability methods into model development will be essential for supporting robust, clinically deployable AI systems.

## Figures and Tables

**Table 3:** Overview of the included studies focusing on classification results.

AUT	FEAT EXT	FEAT	FEAT SEL	VAL APP	EV	AI MODEL	HYPERPARAMETER	CLASSES	PERF	XAI	MAJOR FINDINGS
Maqsood S. et al. (2022) [65]	MobileNetV2	CNN feat	Entropy based method	HO: 70/0/30 5-fold CV	No	Kernel M-SVM	NA	G, M, PT	ACC, SN, SP	No	**BraTS 2018** ACC = 97.47%, SN = 97.22%, SP = 97.94% **BTF** ACC = 98.92%, SN = 98.82%, SP = 99.02%
Mahajan A. et al. (2023) [37]	CNN	CNN + clinical-radiological feat	No	HO: 68/10/22	No	NA	Lfun: Dice loss; ep: 40	EP, MD, BSG, PA	ACC, SP, SN, PPV, NPV	No	BSG: ACC = 94.29%, SN = 88.24%, SP = 96.23%, PPV = 88.24%, NPV = 96.24% EP: ACC = 90.00%, SN = 64.29%, SP = 96.43%, PPV = 81.82%, NPV = 91.53% MD: ACC = 98.57%, SN = 61.90%, SP = 100.00%, PPV = 100.00%, NPV = 85.96% PA: ACC = 84.29%, SN = 100.00%, SP = 78.85%, PPV = 62.07%, NPV = 100.00%
Abd-Ellah, MK et al. (2024) [57]	CNN	CNN feat	No	HO: 75/0/25	No	TRDCNN	ep = 100; Lfun = BCE; opt = SGD; lr = 0.001; Mom = 0.9; Decay = 5.00 × 10^−6^	HGG, LGG	ACC, SN, SP	No	ACC = 98.88%, SP = 99.00%, SN = 98.66%
Rai, HM. et al. (2024) [60]	CNN	CNN feat	No	HO: 87/12/1	No	2D Two-headed UNetEfficientNets	UNetEfficientNets-B0: lr: 0.01 (encoder), 0.001 (decoder); Lfun: Focal + BCE loss; bs: 16; opt (decoder): AdamW UNetEfficientNets-B1: lr: 0.01 (encoder), 0.001 (decoder); Lfun: Focal + BCE; bs: 12; opt (decoder): AdamW UNetEfficientNets-B2: lr: 0.01 (encoder), 0.001 (decoder); Lfun: Focal + BCE; bs: 12; opt (decoder): AdamW UNetEfficientNets-B3: lr: 0.01 (encoder), 0.001 (decoder); Lfun: Focal + BCE; bs: 10; opt (decoder): AdamW UNetEfficientNets-B4: lr: 0.01 (encoder), 0.001 (decoder); Lfun: Focal + BCE; bs: 4; opt (decoder): AdamW UNetEfficientNets-B5: lr: 0.01 (encoder), 0.001 (decoder); Lfun: Focal + BCE; bs: 2; opt (decoder): AdamW	G, M, PT	ACC, SN, SP	No	**UNetEfficientNet-B0** Global: PC = 96.67%, SN = 97.00%, ACC = 94.80% M: PC = 91.00%, SN = 92.00% G: PC = 99.00%, SN = 99.00% PT: PC = 100.00%, SN = 100.00% **UNetEfficientNet-B1** Global: PC = 98.00%, SN = 97.67%, ACC = 99.00% M: PC = 94.00%, SN = 94.00% G: PC = 100.00%, SN = 99.00% PT: PC = 100.00%, SN = 100.00% **UNetEfficientNet-B2** Global: PC = 80.33%, SN = 81.67%, ACC = 93.00% M: PC = 79.00%, SN = 67.00% G: PC = 97.00%, SN = 98.00% PT: PC = 65.00%, SN = 80.00% **UNetEfficientNet-B3** Global: PC = 98.00%, SN = 98.00%, ACC = 96.00% M: PC = 94.00%, SN = 95.00% G: PC = 100.00%, SN = 99.00% PT: PC = 100.00%, SN = 100.00% **UNetEfficientNet-B4** Global: PC = 98.67%, SN = 99.00%, ACC = 99.40% M: PC = 96.00%, SN = 98.00% G: PC = 100.00%, SN = 99.00% PT: PC = 100.00%, SN = 100.00% **UNetEfficientNet-B5** Global: PC = 97.33%, SN = 97.67%, ACC = 99.30% M: PC = 93.00%, SN = 94.00% G: PC = 99.00%, SN = 99.00% PT: PC = 100.00%, SN = 100.00%
Sobhaninia Z. et al. (2023) [61]	CNN	CNN feat	No	5-fold CV	No	Multiscale Cascaded Multitask Network	lr: 0.001, Lfun: Dice loss + BCE; opt: SGD; ep: 150	G, M, PT	ACC	No	**Multiscale Cascaded Multitask Network** ACC = 95.09% **Multiscale Cascaded Multitask Network +** **Aggreagtion Module** Global: ACC = 97.99% M: PC = 97.60% G: PC = 98.32% PT: PC = 97.10%
Aumente-Maestro C. et al. (2025) [18]	Radiomics	MRI, Segmentation feat	No	5-folds CV	No	XGBoost	N estimators: 1000; Max depth: 4; lr: 0.01	HGG, LGG	ACC, bACC, SP, SN, PC, f1-score	Abs rank	**MRI features** ACC = 91.30%, bACC = 83.70%, SN = 96.60%, SP = 70.90%, PC = 93.00%, f1-score = 94.70% **Segmentation features** ACC = 81.30%, bACC = 67.30%, SN = 91.10%, SP = 43.40%, PC = 86.20%, f1-score = 88.50% **All features** ACC = 93.20%, bACC = 87.40%, SN = 97.30%, SP = 77.60%, PC = 94.50%, f1-score = 95.80% **Explainability features** Number of voxels of ET, number of voxels of CT, kurtosis
Krishnasamy N. et al. (2023) [38]	CNN	CNN feat	No	G: HO: 89/0/11 M: HO: 88/0/12 PT: HO: 92/0/8 No tumor: HO: 79/0/21	No	ResNet-50, MobileNet	lr: 0.0001; lr decay: 0.9; ep: 250; opt: Adam; Afun: ReLU; Test Bs: 20	G, M, PT	ACC, PC, SN, SP, f1-score, NPV, PPV	No	**Model 1 (FCN-32 + ResNet-50)** ACC = 98.93%, PC = 96.00%, SN = 94.00%, f1-score = 94.00%, SP = 98.70%, PPV = 79.00%, NPV = 83.00% **Model 2 (SegNet + MobileNet)** ACC = 98.63%, PC = 95.00%, SN = 92.00%, f1-score = 93.00%, SP = 98.50%, PPV = 87.00%, NPV = 86.00%
Zaitoon R. et al. (2023) [43]	CNN	CNN feat	No	HO: 70/10/20	No	DBT-CNN	bs: 256, Layers: 3	HGG, LGG	ACC, SN, PC, SP, f1-score, AUC	No	**DBT-CNN** HGG: ACC = 98.51 ± 0.18%, SN = 97.23 ± 0.28%, PC = 99.34 ± 0.23%, SP = 98.62 ± 0.46%, f1-score = 97.49 ± 0.46%, AUC = 99.65 ± 0.02% LGG: ACC = 99.28 ± 0.18%, SN = 97.83 ± 1.22%, PC = 98.56 ± 1.26%, SP = 98.88 ± 0.16%, f1-score = 97.68 ± 1.64%, AUC = 99.02 ± 0.01% HGG NCR: ACC = 98.42 ± 0.08%, SN = 96.22 ± 1.30%, PC = 98.24 ± 0.10%, SP = 98.96 ± 0.30%, f1-score = 97.22 ± 0.10%, AUC = 99.00% HGG ED: ACC = 99.15 ± 0.03%, SN = 97.09 ± 1.64%, PC = 98.29 ± 0.16%, SP = 99.64 ± 0.05%, f1-score = 98.09 ± 0.23%, AUC = 99.00% HGG ET: ACC = 98.68 ± 0.12%, SN = 95.15 ± 2.04%, PC = 98.69 ± 0.11%, SP = 98.68 ± 0.12%, f1-score = 98.12 ± 0.34%, AUC = 99.00% HGG NET: ACC = 99.35 ± 0.01%, SN = 96.02 ± 2.09%, PC = 99.35 ± 0.01%, SP = 98.66 ± 0.08%, f1-score = 98.28 ± 0.27%, AUC = 99.00% LGG NCR: ACC = 98.34 ± 0.13%, SN = 97.29 ± 1.55%, PC = 97.13 ± 0.39%, SP = 99.23 ± 0.04%, f1-score = 98.49 ± 0.51%, AUC = 98.00% LGG ED: ACC = 98.93 ± 0.10%, SN = 97.86 ± 0.29%, PC = 98.52 ± 0.28%, SP = 99.19 ± 0.11%, f1-score = 98.33 ± 0.29%, AUC = 99.00% LGG ET: ACC = 99.26 ± 0.07%, SN = 97.43 ± 1.61%, PC = 98.43 ± 0.14%, SP = 99.72 ± 0.13%, f1-score = 98.64 ± 0.33%, AUC = 99.00% LGG NET: ACC = 99.12 ± 0.04%, SN = 98.30.36%, PC = 98.27 ± 0.23%, SP = 98.27 ± 0.22%, f1-score = 98.73 ± 0.13%, AUC = 97.00%

Legend: AUT: authors, FEAT EXT: feature extraction, FEAT: features, FEAT SEL: feature selection, VAL APP: validation approach, EV: external validation, AI: artificial intelligence, PERF: performance, XAI: Explainable artificial intelligence, NA: Not Available, MRI: magnetic resonance imaging, M-SVM: Multiclass Support Vector Machine, SP: Specificity, SN: Sensitivity, PC: Precision, ACC: Accuracy, SGD: Stochastic Gradient Descent, HGG: High-Grade Glioma, G: glioma, M: meningioma, PT: pituatory tumor, EP: Ependymoma, MD: medulloblastoma, BSG: brainstem glioma, PA: pilocytic astrocytoma, CV: cross-validation, HO: hold-out, Abs rank: absolute ranking, lr: learning rate, opt: optimizer, bs: batch size, ep: epochs, Lfun: loss function, Afun: activation function, Mom: momentum, PPV: Positive Predictive Value DBT-CNN: Deep Brain Tumor Convolutional Neural Network, AUC: Area Under the Curve.

## Data Availability

No new data were created or analyzed in this study. Data sharing is not applicable.

## Supplementary Materials

The following supporting information can be downloaded at: https://www.mdpi.com/article/10.3390/brainsci16050468/s1, PRISMA 2020 Checklist. Table S1. Definition of performance metrics used in the segmentation task to evaluate DL models. Table S2. Definition of performance metrics used in the classification task to evaluate DL models.
