# Supplementary material for: Advancing Brain Tumor Diagnosis Using Deep Learning: A Systematic and Critical Review on Methodological Approaches to Glioma Segmentation and Classification Through Multiparametric MRI
Source: Brain Sci. 2026 Apr 27;16(5):468. doi: 10.3390/brainsci16050468 (PMC13204197; doi:10.3390/brainsci16050468)
Supplement: Supplementary file 1 [file brainsci-16-00468-s001.zip › brainsci-4232379-supplementary.pdf]

**Table S1.** Definition of performance metrics used in the segmentation task to evaluate DL models

| Performance metrics | Formula | Definition |
|---|---|---|
| Dice Score Coefficient (DSC) | $\frac{2\|A \cap B\|}{\|A\| + \|B\|}$ | The DSC quantifies the overlap between the automatic segmentation (A) and the manual reference standard (B). From a clinical perspective, the DSC reflects how accurately the model-derived segmentation matches the true lesion or anatomical structure; higher values indicate a more precise estimation of both lesion size and spatial localisation. |
| Intersection over Union (IoU) | $\frac{\|A \cap B\|}{\|A \cup B\|}$ | The IoU, also known as the Jaccard Index, quantifies the overlap between the automatic segmentation (A) and the manual reference standard (B) as the ratio of their intersection to their union. From a clinical perspective, IoU provides a more stringent assessment of segmentation accuracy, reflecting how well the predicted lesion or anatomical structure aligns with the true one; higher values indicate a more precise delineation with fewer false positives (FP) and false negatives (FN). |
| Accuracy | $\frac{TP + TN}{TP + FP + TN + FN}$ | Accuracy measures the proportion of correctly classified voxels between the automatic segmentation (A) and the manual reference standard (B), considering both lesion and non-lesion regions. From a clinical perspective, accuracy reflects the overall agreement between model predictions and ground truth. |
| Balanced Accuracy | $\frac{1}{2}\left(\frac{TP}{TP + FN} + \frac{TN}{TN + FP}\right)$ | Balanced accuracy measures the average of sensitivity (TPR) and specificity (TNR) between the automatic segmentation (A) and the manual reference standard (B). From a clinical perspective, it provides a more reliable evaluation of segmentation performance under class imbalance. |
| Matthews Correlation | $\frac{(TP * TN) - (FP * FN)}{\sqrt{(TP + FP)(TP + FN)(TN + FP)(TN + FN)}}$ | The MCC evaluates the quality of binary segmentation by considering |

| | | |
|---|---|---|
| Coefficient (MCC) | | true positives, true negatives, false positives, and false negatives in a single metric. From a clinical perspective, MCC provides a balanced assessment of segmentation performance even in the presence of class imbalance; higher values indicate a more reliable identification of both lesion and non-lesion regions, which is particularly relevant in conditions with sparse abnormalities. |
| f1-score | $\frac{2 * TP}{2 * TP + FP + FN}$ | The f1-score is the harmonic mean of precision and recall, measuring the balance between false positives (FP) and false negatives (FN) in the segmentation. From a clinical perspective, it reflects how well the model identifies lesion voxels while minimising both missed lesions and over-segmentation; higher values indicate a better trade-off between sensitivity and precision. |
| f2-score | $\frac{5 * TP}{5 * TP + 4 * FN + FP}$ | The f2-score is a weighted version of the f-measure that emphasises recall over precision, assigning greater weight to false negatives (FN). From a clinical perspective, it is particularly relevant in scenarios where missing lesions are more critical than over-segmentation; higher values indicate improved sensitivity in detecting pathological regions, even at the expense of increased false positives (FP). |
| 95th percentile Hausdorff Distance (HD95) | $max(h_{95}(A,B), h_{95}(B,A))$ | The HD95 between the automatic segmentation (A) and the manual reference standard (B) is defined as the maximum of the 95th percentile of the directed distances $h_{95}(A,B)$ and $h_{95}(B,A)$. From a clinical perspective, HD95 quantifies the boundary discrepancy between the two segmentations while reducing the influence of extreme outlier errors; lower values indicate a closer agreement in lesion contour and spatial localisation. |

| | | |
|---|---|---|
| k-Cohen | $\frac{p_o - p_e}{1 - p_e}$ | Cohen's k measures the agreement between the automatic segmentation (A) and the manual reference standard (B), accounting for chance agreement, and is defined as the observed agreement ($p_o$) divided by the expected agreement by chance ($p_e$). From a clinical perspective, κ reflects the consistency between model predictions and expert annotations beyond random coincidence; higher values indicate stronger agreement and greater reliability of the segmentation in supporting clinical decision-making |
| Sensitivity | $\frac{TP}{TP + FN}$ | Sensitivity measures the proportion of true lesion voxels correctly identified by the model among all actual lesion voxels. From a clinical perspective, it reflects the ability to detect pathological regions; higher values indicate fewer missed lesions. |
| Specificity | $\frac{TN}{TN + FP}$ | Specificity measures the proportion of correctly identified non-lesion voxels among all actual non-lesion voxels. From a clinical perspective, it reflects the model's ability to avoid false positives; higher values indicate more accurate identification of healthy tissue and reduced over-segmentation. |
| Precision | $\frac{TP}{TP + FP}$ | Precision measures the proportion of predicted lesion voxels that are truly lesions. From a clinical perspective, it reflects the reliability of positive predictions; higher values indicate that detected lesions are more likely to correspond to true pathological regions, reducing false alarms. |
| Area under the ROC (AUC) | $\int_0^1 TPR(FPR)d(FPR)$ | The AUC summarises the model's ability to discriminate between lesion and non-lesion voxels across all possible classification thresholds. From a clinical perspective, AUC reflects the model's overall diagnostic performance; higher values indicate a better trade-off between sensitivity and specificity, independent of the chosen decision threshold. |

| | | |
|---|---|---|
| Tversky index (TI) | $\frac{TP}{TP + \alpha FP + \beta FN}$ | The TI is a generalisation of overlap-based metrics that introduces weighting factors for false positives and false negatives through parameters alpha and beta. From a clinical perspective, it allows tuning the segmentation evaluation according to the relative importance of over-segmentation and missed lesions; higher values indicate better agreement with the reference standard. |

**Table S2.** Definition of performance metrics used in the classification task to evaluate DL models

| **Performance metrics** | **Formula** | **Definition** |
|---|---|---|
| Accuracy | $\frac{TP + TN}{TP + FP + TN + FN}$ | From a clinical perspective, it reflects the overall diagnostic correctness of the model; however, in imbalanced datasets (e.g., low disease prevalence), high accuracy may be driven by the correct classification of healthy subjects rather than true disease detection. |
| Balanced accuracy | $\frac{1}{2}(\frac{TP}{TP + FN} + \frac{TN}{TN + FP})$ | From a clinical perspective, it provides a more reliable estimate of diagnostic performance by equally weighting the model's ability to detect both diseased and healthy subjects. |
| Sensitivity | $\frac{TP}{TP + FN}$ | Sensitivity measures the proportion of diseased patients that the model correctly identifies. From a clinical perspective, it reflects the ability to detect the condition of interest; higher values indicate fewer missed diagnoses, which is particularly critical in screening settings. |
| Specificity | $\frac{TN}{TN + FP}$ | Specificity measures the proportion of healthy subjects correctly classified. From a clinical perspective, it reflects the ability to avoid false positive diagnoses; higher values reduce unnecessary follow-up tests, patient anxiety, and healthcare burden. |
| Positive Predictive Value (PPV) | $\frac{TP}{TP + FP}$ | The PPV measures the proportion of predicted positive cases that are truly positive. From a clinical perspective, PPV reflects the reliability of positive predictions; higher values indicate that detected lesions are more likely to correspond to true pathological findings, reducing the rate of false |

| | | |
|---|---|---|
| | | positives and unnecessary clinical concern. |
| Negative Predictive Value (NPV) | $\frac{TN}{TN + FN}$ | The NPV measures the proportion of predicted negative cases that are truly negative. From a clinical perspective, NPV reflects the reliability of negative predictions; higher values indicate that regions classified as healthy are indeed free of pathology, which is essential for confidently ruling out disease. |
| Precision | $\frac{TP}{TP + FP}$ | Precision measures the proportion of subjects predicted as diseased who truly have the condition. From a clinical perspective, it reflects the reliability of a positive test result; higher values indicate that a positive prediction is more likely to correspond to a true diagnosis. |
| f1-score | $\frac{2 * TP}{2 * TP \; + \; FP \; + FN}$ | The f1-score is the harmonic mean of precision and sensitivity, balancing false positives and false negatives. From a clinical perspective, it provides a single measure of diagnostic performance when both missed diagnoses and false alarms are clinically relevant. |
| Area under the ROC (AUC) | $\int_0^1 TPR(FPR)d(FPR)$ | The AUC quantifies the model's ability to discriminate between diseased and healthy subjects across all possible decision thresholds. From a clinical perspective, it reflects the intrinsic diagnostic capability of the model, independent of a specific cutoff, and is particularly useful for comparing models or selecting optimal thresholds for clinical deployment. |
